# Heterogeneity of G protein activation by the calcium-sensing receptor

**DOI:** 10.1530/JME-21-0058

**Published:** 2021-06-02

**Authors:** Hasnat Ali Abid, Asuka Inoue, Caroline M Gorvin

**Affiliations:** 1 Institute of Metabolism and Systems Research and Centre for Endocrinology, Diabetes and Metabolism, University of Birmingham, Birmingham, UK; 2 Graduate School of Pharmaceutical Sciences, Tohoku University, Sendai, Miyagi, Japan; 3 Centre of Membrane Proteins and Receptors (COMPARE), Universities of Birmingham and Nottingham, UK

**Keywords:** calcium homeostasis, calcium signalling, G protein-coupled receptor, parathyroid and bone, signal transduction

## Abstract

The calcium-sensing receptor (CaSR) is a G protein-coupled receptor that plays a fundamental role in extracellular calcium (Ca^2+^_e_) homeostasis by regulating parathyroid hormone release and urinary calcium excretion. The CaSR has been described to activate all four G protein subfamilies (Gα_q/11_, Gα_i/o_, Gα_12/13_, Gα_s_), and mutations in the receptor that cause hyper/hypocalcaemia, have been described to bias receptor signalling. However, many of these studies are based on measurements of second messengers or gene transcription that occurs many steps downstream of receptor activation and can represent convergence points of several signalling pathways. Therefore, to assess CaSR-mediated G protein activation directly, we took advantage of a recently described NanoBiT G protein dissociation assay system. Our studies, performed in HEK293 cells stably expressing CaSR, demonstrate that Ca^2+^_e_ stimulation activates all Gα_q/11_ family and several Gα_i/o_ family proteins, although Gα_z_ was not activated. CaSR stimulated dissociation of Gα_12/13_ and Gα_s_ from Gβ-subunits, but this occurred at a slower rate than that of other Gα-subunits. Investigation of cDNA expression of G proteins in three tissues abundantly expressing CaSR, the parathyroids, kidneys and pancreas, showed Gα_11_, Gα_z_, Gα_i1_ and Gα_13_ genes were highly expressed in parathyroid tissue, indicating CaSR most likely activates Gα_11_ and Gα_i1_ in parathyroids. In kidney and pancreas, the majority of G proteins were similarly expressed, suggesting CaSR may activate multiple G proteins in these cells. Thus, these studies validate a single assay system that can be used to robustly assess CaSR variants and biased signalling and could be utilised in the development of new pharmacological compounds targeting CaSR.

## Introduction

The calcium-sensing receptor (CaSR) is a class C homo-dimeric G protein-coupled receptor (GPCR) that plays a fundamental role in extracellular calcium (Ca^2+^_e_) homeostasis by regulating parathyroid hormone (PTH) release and urinary calcium excretion. Consistent with its critical role in maintaining serum calcium concentrations, the CaSR is highly expressed on the surface of parathyroid and kidney cells (Riccardi & Brown 2010). The CaSR is also widely expressed on other tissues, including significant expression on pancreatic islet α- and β-cells (Regard *et al.* 2008), and has been described to mediate a diverse range of functions, including inflammation, bronchoconstriction, gastrointestinal hormone secretion and glucose metabolism (Hofer *et al.* 2000, Rossol *et al.* 2012, Yarova *et al.* 2015, Zietek & Daniel 2015).

The importance of CaSR in the regulation of Ca^2+^_e_ is highlighted in patients with germline mutations of the receptor that present with disorders of calcium homeostasis. Inactivating mutations cause familial hypocalciuric hypercalcaemia type-1 (FHH1), characterised by lifelong elevated serum calcium, moderate-to-high PTH concentrations, and low renal calcium excretion, and rarely cause neonatal severe hyperparathyroidism, which can be fatal if untreated (Pollak *et al.* 1993). Activating CaSR mutations cause autosomal dominant hypocalcaemia type-1 (ADH1), characterised by mild-to-moderate hypocalcaemia, with inappropriately low-to-normal serum PTH (Pearce *et al.* 1996). In addition, inactivating mutations in the G protein-α11 (Gα11), by which the CaSR signals and the adaptor protein-2 σ-subunit (AP2σ), which regulates endocytosis, cause FHH2 and FHH3, respectively; whereas activating Gα11 mutations cause ADH2 (Mannstadt *et al.* 2013, Nesbit *et al.* 2013*a*,*b*).

On stimulation by elevations in Ca^2+^_e_, the CaSR activates diverse signalling pathways. In most cell types, CaSR is understood to signal via Gα_q/11_ to activate phospholipase C (PLC)-mediated increases in intracellular calcium and mitogen-activated protein kinase (MAPK), and Gα_i/o_ to inhibit adenylate cyclase and reduce cAMP (Kifor *et al.* 1997, Brown & MacLeod 2001, Hofer & Brown 2003). Additionally, CaSR has been described to activate cytoskeletal remodelling, attributed to Gα_12/13_ pathways in some cell types (e.g. MDCK cell-lines) (Huang *et al.* 2004) and Gα_q/11_ in others (Pi *et al.* 2002). Finally, in breast cancer and AtT20 pituitary tumour cells, CaSR activates elevations in cAMP by switching of CaSR coupling from Gα_q/11_ and Gα_i/o_ to Gα_s_ (Mamillapalli *et al.* 2008, Mamillapalli & Wysolmerski 2010).

The specific coupling of CaSR to individual G protein pathways was investigated in early studies by radio-ligand measurements of phospholipases; while subsequent studies have measured a range of second messengers in cells depleted of Gα proteins by chemical (e.g. YM-254890 or pertussis toxin that inhibit G_q__/11_ and G_i__/o_, respectively) or genetic manipulation (by siRNA knockdown or CRISPR-Cas9) (Kifor *et al.* 1997, Pi *et al.* 2002, Gorvin *et al.* 2018*d*). Although these studies have successfully identified many components of the CaSR signalling pathway, these methodologies are associated with several disadvantages, including variable sensitivity and complexity of assays; poor targeting of individual G proteins by chemical inhibitors and siRNAs, and the available CRISPR-generated cell-lines have deletions of whole G protein subfamilies rather than individual G proteins (Takasaki *et al.* 2004, Devost *et al.* 2017, Gundry *et al.* 2017). These limitations, combined with the distinct activation kinetics of second messengers, and recognition that multiple G proteins converge on the same signal proteins or genes (Goldsmith & Dhanasekaran 2007), impair the researcher’s ability to compare GPCR stimulation of different signalling pathways using many assay platforms.

The ability to assess GPCR activation of individual G proteins is increasingly important as it is recognised that receptors may couple preferentially to different signalling pathways in diverse tissues, and disease-causing mutations may confer alternative receptor conformations that couple differentially to intracellular signalling pathways (Wingler & Lefkowitz 2020). Such tissue- and mutation-specific bias has been described for the CaSR indicating that assessment of multiple signalling pathways may be required to assess pathogenicity of receptor variants identified in patient samples (Leach *et al.* 2012, Gorvin *et al.* 2018*c*). Moreover, allosteric modulators of the CaSR exhibit signalling bias, indicating it may be possible to develop additional pharmacological compounds that convey unique functional selectivity on individual G proteins to separate desirable from adverse (i.e. off-target) effects (Hauser *et al.* 2017). Therefore, a direct measure of G protein coupling to CaSR would be beneficial to aid in future drug design, in assigning decisions regarding the pathogenicity of receptor variants, and in identifying biased signalling by receptor mutants.

Recently, a NanoBiT G protein dissociation assay system that assesses activation of multiple Gα subunits was described (Inoue *et al.* 2019). This assay utilises a split nano-luciferase (NanoLuc), in which the large part of NanoLuc (LgBiT) is tagged to the Gα-subunits and the small part (SmBiT) is tagged to the Gβ-subunit. Thus G protein activation can be monitored by agonist-induced reductions in luciferase due to separation of the Gα- and Gβγ-subunits following receptor stimulation. The development of this assay afforded an opportunity to interrogate the promiscuity of CaSR coupling to G proteins without the problem of signal pathway cross-talk. These studies, combined with gene expression analyses, indicate that the physiological effects of CaSR are likely mediated by a combination of G protein activation and expression of individual Gα-proteins in different tissues. This simple assay system is easily scalable, measures in real-time and could be used for drug screening and assessment of pathogenicity and signalling bias of receptor variants by multiple CaSR signalling pathways.

## Methods

### Cell culture and transfection

Adherent HEK293 (AdHEK) cells were purchased from Agilent Technologies. HEK293 cells with deletion of the Gα_q/11_, Gα_i/o_, Gα_12/13_ or Gα_s/l_ family of G proteins (G protein knockout cells) were described previously (Inoue *et al.* 2019). AdHEK cells and G protein knockout cells were maintained in DMEM-Glutamax media (Sigma) with 10% foetal bovine serum (FBS, Sigma) at 37°C, 5% CO_2_. The full-length CaSR cDNA with an N-terminal FLAG tag was PCR amplified (reagents from Promega) from total human kidney cDNA (Ambion) and cloned into a pcDNA3.1 expression vector (Life Technologies). The full-length somatostatin receptor-5 (SSTR5) was PCR amplified from SSTR5-Tango (a gift from Bryan Roth (Addgene plasmid # 66506; RRID:Addgene_66506) (Kroeze *et al.* 2015)) and cloned into pcDNA3.1. Expression constructs were sequence-verified by Source Bioscience (Nottingham, UK). NanoBiT G protein dissociation and the IP3 biosensor constructs were described in detail previously (Inoue *et al.* 2019). The pGloSensor-20F plasmid was purchased from Promega. Transfections were performed using Lipofectamine 2000 (LifeTechnologies), according to the manufacturer’s instructions. To generate AdHEK-CaSR, cells stably transfecting pcDNA3.1-FLAG-CaSR cells were plated in six-well plates, transfected with 1 µg DNA, then single clones selected using cell culture media containing 500 µg/mL geneticin (Gibco).

### Western blot analysis

Western blot analysis was performed as previously described (Gorvin *et al.* 2018*d*). Endogenous calnexin was used as a loading control. Lysates were resuspended in Laemmli buffer, boiled and separated on 6% SDS-PAGE gels. Following transfer to polyvinylidene difluoride membrane (ThermoFisher), blots were blocked in 5% marvel/TBS-T, then probed with anti-CaSR (ADD, Abcam), anti-Gα11 (SantaCruz Biotechnology), anti-Gα12 (SantaCruz), and anti-calnexin (Millipore) antibodies. Blots were visualised using the Immuno-Star WesternC kit (BioRadK) on a BioRad Chemidoc XRS+ system. For cell surface expression of CaSR, plasma membrane fractions were extracted using the Plasma Membrane Protein Extraction kit (Abcam). Plasma membrane calcium adenosine triphosphatase (PMCA1) (Abcam) was used as a housekeeping protein for the plasma membrane fraction.

### cAMP GloSensor assays

Cells were plated in six-well plates and transfected with 50 ng pGloSensor-20F plasmid. Twenty-four hours later cells were re-plated in 96-well plates in FluoroBrite DMEM media (ThermoScientific) containing 10% FBS. On the following day cells were incubated with 100 µL of equilibration media consisting of Hank’s buffered saline solution (HBSS, calcium and magnesium-free) containing 2% (v/v) dilution of the GloSensor cAMP Reagent stock solution according to manufacturer’s instructions. Cells were incubated for 2 h at 37°C. Basal luminescence was read on a PheraStar FSX (BMGLabtech) for 8 min, then agonist added with 10 µM forskolin (Sigma), and plates read for a further 30 min.

### NanoBiT G protein dissociation and IP3 assays

Cells were plated in six-well plates and transfected 24 h later with: 250 ng LgBiT-Gα plasmid, 500 ng SmBiT-Gβ plasmid, 500 ng untagged Gγ_2_ plasmid. For studies shown in Supplementary Figs 1, 2 and 3 (see section on supplementary materials given at the end of this article), AdHEK were also transiently transfected with 250 ng of FLAG-CaSR, while studies of SSTR5 were transiently transfected with pcDNA3.1-SSTR5. For reactions with LgBiT-Gα_11_ cells were co-transfected with RIC8A, as previously described (Inoue *et al.* 2019). Following 24 h, cells were harvested in DMEM-fluorobrite (ThermoScientific) with 10% FCS and 4mM L-glutamine and seeded in 8 wells of a 96-well plate. NanoBiT assays were performed the following day using NanoGlo reagent (Promega). Media was changed to HBSS without antibiotics (containing 0.1 mM CaCl_2_) on the morning of the assay. Approximately 4 h later, each well was loaded with 40 µL substrate and baseline signal read on a Pherastar FSX plate reader (BMGLabtech, Aylesbury, UK) at 37°C for four cycles (equivalent to 8 min). Vehicle and agonists were prepared in Hank’s Balanced Saline Solution (HBSS, Sigma) at 10× concentration and added to wells once baselines were stable and responses recorded immediately following agonist addition for up to 25 min. All responses were normalised to that treated with vehicle at 0 min. For SSTR5 studies cells were exposed to either vehicle (DMSO) or 50 nM somatostatin (purchased from Sigma). The consequent fold-change values were fitted to a four-parameter sigmoidal concentration-response using GraphPad Prism. IP3 biosensor assays were performed similarly using 200 ng LgBiT-IP3R2-SmBiT plasmid. For IP3 measurements, basal luminescence was read on a PheraStar FSX (BMGLabtech) for 8 min, then agonist added, and plates read for a further 30 min.

### Gene expression analysis

Analysis of gene expression in parathyroid, kidney and pancreatic tissues was performed using the Gene Expression Omnibus (GEO) website (https://www.ncbi.nlm.nih.gov/geo/) using the search term ‘parathyroid’, ‘kidney’, ‘pancreas’ and ‘human’, accession date August 2020. Two datasets, #GSE83421 (Balenga *et al.* 2017) and #GSE2193 (Shyamsundar *et al.* 2005), were identified and used in analyses using GEO2R for parathyroid tissue. These comprised data from 9 normal parathyroids and 25 adenoma tissues from patients with primary hyperparathyroidism. For kidney and pancreas expression the following datasets were used: GSM12660, GSM12661, GSM12663, GSM12659, GDS181, GDS1085, GDS1663 (Su *et al.* 2002, Shyamsundar *et al.* 2005). Gene expression values for a panel of housekeeping genes: actin, beta (*ACTB*); phosphoglycerate kinase 1 (*PGK1*); peptidylprolyl isomerase A (*PPIA*); beta-2-microglobulin (*B2M*); transferrin receptor (*TFRC*); glucuronidase, beta (*GUSB*); hypoxanthine phosphoribosyltransferase 1 (*HPRT1*); TATA box binding protein (*TBP*); and tubulin, beta (TUBB) were acquired and the mean value used for normalisation using Microsoft Excel 2013. Expression of each G protein subunit was expressed relative to the geometric mean of the housekeeping genes in each sample and plotted using GraphPad Prism 7. A cut-off of representation in at least four datasets was used for analysis of expression between genes. To compare expression within each G protein subfamily, the relative expression of each gene was expressed as a fold-change to a representative gene (*GNA11, GNAI1, GNA12, GNAS*). Statistical analyses were performed using ANOVA between two or more datasets and correction for multiple testing applied. A value of *P* < 0.05 was considered significant. Comparisons between the gene expression data and proteome datasets could not be made as there is insufficient proteomic data available on parathyroid tissues.

## Results

### CaSR can couple to all members of the G_q__/11_ subfamily

Previous studies have indicated that CaSR is able to couple promiscuously to multiple G proteins (Kifor *et al.* 1997, Brown & MacLeod 2001, Hofer & Brown 2003, Huang *et al.* 2004). However, these studies are largely based on measurements of second messengers or changes in gene transcription, which can only indicate the G protein subfamily and not necessarily the specific G protein activated. Furthermore, as many signalling pathways share downstream signal components, it may be difficult to differentiate the effects of different G proteins. We therefore used an unbiased approach to determine which specific G proteins are activated by CaSR using a recently described NanoBiT G protein dissociation assay system (Inoue *et al.* 2019). NanoBiT utilises a split nano-luciferase (NanoLuc) in which the large part of NanoLuc (LgBiT) is tagged to the Gα subunits and the small part (SmBiT) is tagged to the Gβ-subunit. Under basal conditions, the heterotrimeric Gαβγ protein is stable and the LgBiT and SmBiT are associated with yielding high levels of luciferase. On activation of the GPCR, Gα and Gβγ dissociate and luciferase levels reduce.

To investigate G protein activation by the CaSR, we first assessed Gα-Gβ3 dissociation with six different concentrations of Ca^2+^_e_ in adherent HEK293 (AdHEK) transiently expressing the CaSR. Robust concentration-dependent increases in G protein dissociation, including within the physiological range of Ca^2+^_e_ (~1–3 mM), were observed for several members of the Gα_q/11_ and Gα_i/o_ families (Supplementary Fig. 1). However, G protein dissociation was only observed for some G proteins (Gαi2, Gαi3, Gα12) at concentrations >3 mM Ca^2+^_e_. Therefore, for subsequent studies, we used 5 mM Ca^2+^_e_, as this concentration was the closest to the physiological range that would allow us to assess dissociation of the largest number of G proteins. G protein dissociation in response to 5 mM Ca^2+^_e_ was not observed in AdHEK cells lacking the CaSR (Supplementary Fig. 2). Overexpression of other untagged Gα proteins did not affect G protein coupling of NanoBiT constructs (Supplementary Fig. 3), nor did deletion of other Gα proteins (Supplementary Fig. 4).

**Figure 1:**
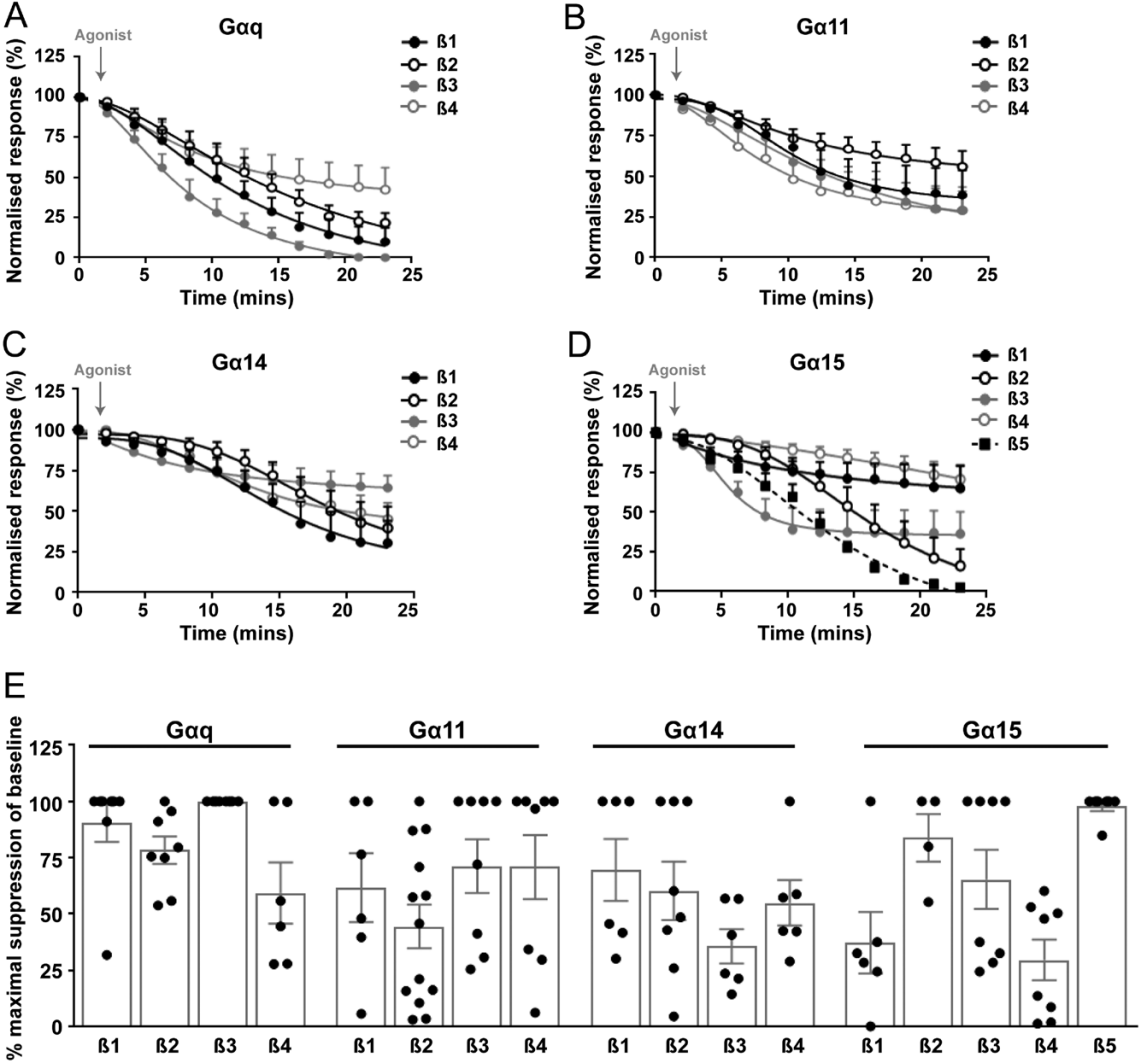
CaSR activation of the G_q/11_ family by NanoBiT G protein dissociation assays. NanoBiT dissociation assays of AdHEK-CaSR cells transiently transfected with: (A) LgBiT-Gα_q_, (B) LgBiT-Gα_11_, (C) LgBiT-Gα_14_, (D) LgBiT-Gα_15_ with SmBiT-Gβ subunits (Gβ1–5) and unlabelled Gγ2 following treatment with 5 mM Ca^2+^_e_. Pre-calcium baselines are not shown. The first baseline value is shown. (E) Percentage suppression of baseline from A, B, C and D. Statistical analyses comparing values of all Gα-Gβ dissociations are shown in Supplementary Table 1. For all panels only those Gα and Gβ pairs that yielded relative luminescence units (RLU) over a threshold of 1 × 10^3^ (i.e. above background luminescence) are shown. All responses were normalised to those under basal conditions (0.1 mM Ca^2+^_e_). Dissociation curves for 5 mM Ca^2+^_e_ are shown (curves with 0.1 mM Ca^2+^_e_ are shown in Supplementary Fig. 7). Curves show mean±s.e.m. for *n* = 6–11 independent assays.

To assess G protein coupling and dissociation between 12 Gα and 5 Gβ subunits we generated a AdHEK cell-line stably expressing the CaSR with an N-terminal FLAG tag (Supplementary Fig. 5). These cells were demonstrated to overexpress CaSR and to activate IP3 accumulation and cAMP depletion on stimulation with increasing doses of Ca^2+^_e_ (Supplementary Fig. 5). All subsequent assays were performed in one clone of this cell-line (clone A, named AdHEK-CaSR). Overexpression of NanoBiT constructs had no effect on CaSR total or cell surface expression, nor on the expression of other G proteins (Supplementary Fig. 6). Assays were performed with the 12 available Gα-LgBiT-subunits in combination with 5 Gβ-SmBiT-subunits. Cells were exposed to low concentrations of Ca^2+^_e_ (0.1 mM) or an activating concentration of 5 mM Ca^2+^_e_. The Gα_q/11_ family was first investigated as *GNA11* is known to be highly expressed in parathyroid tissue and mutations in this gene cause FHH2 and ADH2 (Mannstadt *et al.* 2013, Nesbit *et al.* 2013*a*). The four Gα proteins (q, 11, 14, 15) were assessed with all Gβ-subunits. Cells in which the relative luminescence units (RLU) were below 1000 were excluded as this value was not significantly greater than background luminescence (Supplementary Table 1). Analyses of the G_q__/11_ family demonstrated that all four α-subunits could be activated following exposure of cells to 5 mM Ca^2+^_e_ (Fig. 1A, B, C, D and Supplementary Fig. 7). CaSR produced greater activation responses with Gα_q_ and Gα_11_ than the other family members (Fig. 1A, B, C, D, E and Supplementary Table 2), and therefore it is more likely that these proteins are activated by the receptor when present in CaSR expressing tissues.

### CaSR activates several members of the G_i/o_ subfamily but not Gα_z_

**Figure 2:**
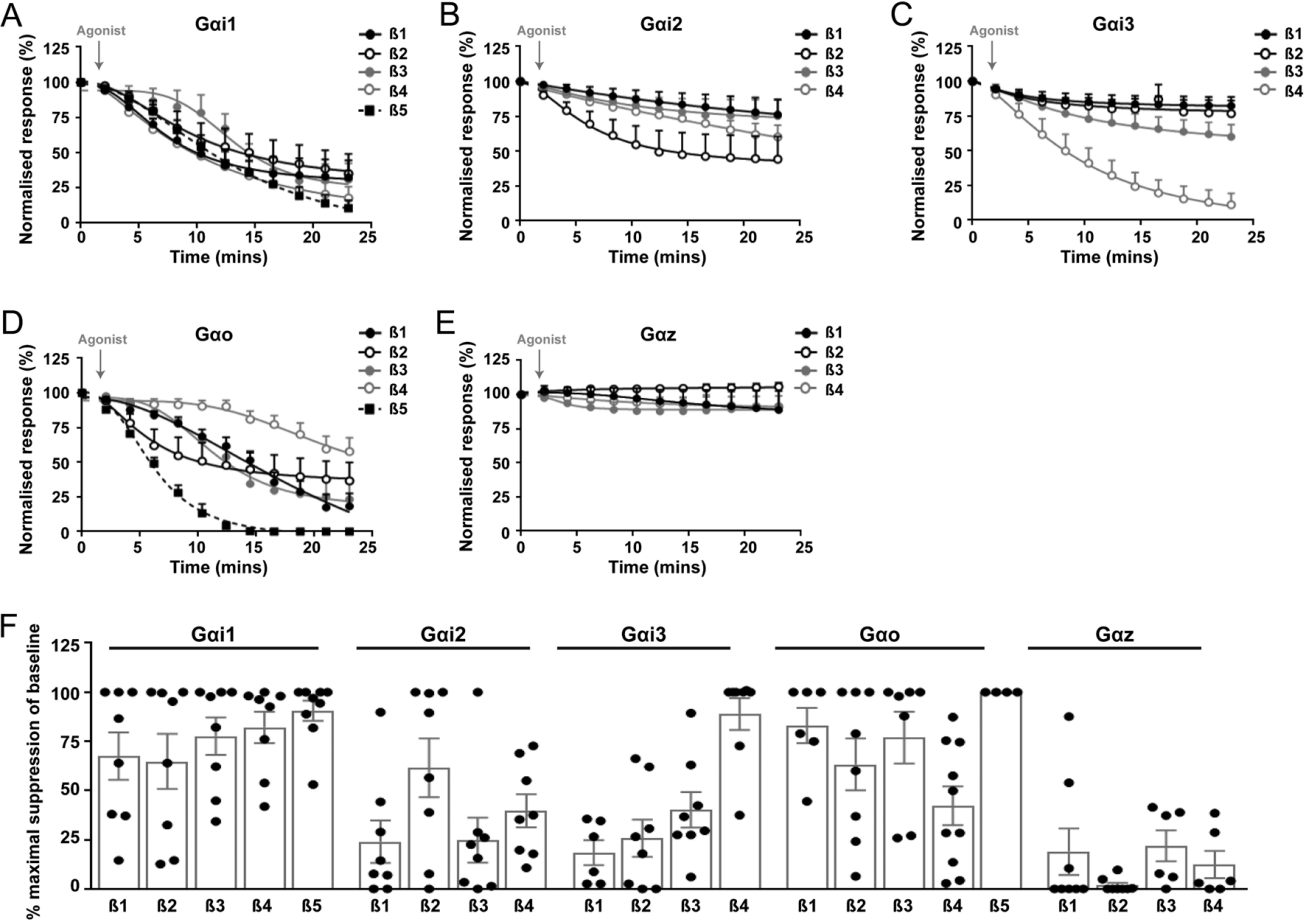
CaSR activation of the G_i/o_ family by NanoBiT G protein dissociation assays. NanoBiT dissociation assays of AdHEK-CaSR cells transiently transfected with: (A) LgBiT-Gα_i1_, (B) LgBiT-Gα_i2_, (C) LgBiT-Gα_i3_, (D) LgBiT-Gα_o_ and (E) LgBiT-Gα_z_ with SmBiT-Gβ subunits (Gβ1–5) and unlabelled Gγ2 following treatment with 5mM Ca^2+^_e_. Pre-calcium baselines are not shown. The first baseline value is shown. (F) Percentage suppression of baseline from A, B, C, D and E. Statistical analyses comparing values of all Gα-Gβ dissociations are shown in Supplementary Table 1. For all panels only those Gα and Gβ pairs that yielded relative luminescence units (RLU) over a threshold of 1 × 10^3^ (i.e. above background luminescence) are shown. All responses were normalised to those under basal conditions (0.1 mM Ca^2+^_e_). Dissociation curves for 5 mM Ca^2+^_e_ are shown (curves with 0.1 mM Ca^2+^_e_ are shown in Supplementary Figure 8). Curves show mean ± s.e.m. for *n* = 6–10 independent assays.

CaSR has been described to reduce cAMP in many studies, and signalling by these pathways has been demonstrated to play a role in PTH secretion in parathyroid cells (Brown *et al.* 1978). Five members of the G_i__/o_ subfamily were investigated by NanoBiT dissociation assays. CaSR robustly activated Gα_i1_ when combined with any Gβ-subunit, and Gα_o_ similarly activated all Gβ-subunits, although activation, when combined with β4, was slow (Fig. 2 and Supplementary Fig. 8). Gα_i2_ and Gα_i3_ were activated only when combined with one Gβ-subunit and Gα_z_ was not activated at all (Fig. 2). This was not due to inactivity of the LgBiT-Gα_z_ plasmid as other GPCRs could activate this G protein (Supplementary Fig. 9). Thus, activation of the G_i__/o_ family in CaSR expressing cells is unlikely to occur by Gα_z_ and activation of some family members (Gα_i2_ and Gα_i3_) will be dependent on β-subunit expression.

### Other G protein families are activated slowly by the CaSR

**Figure 3:**
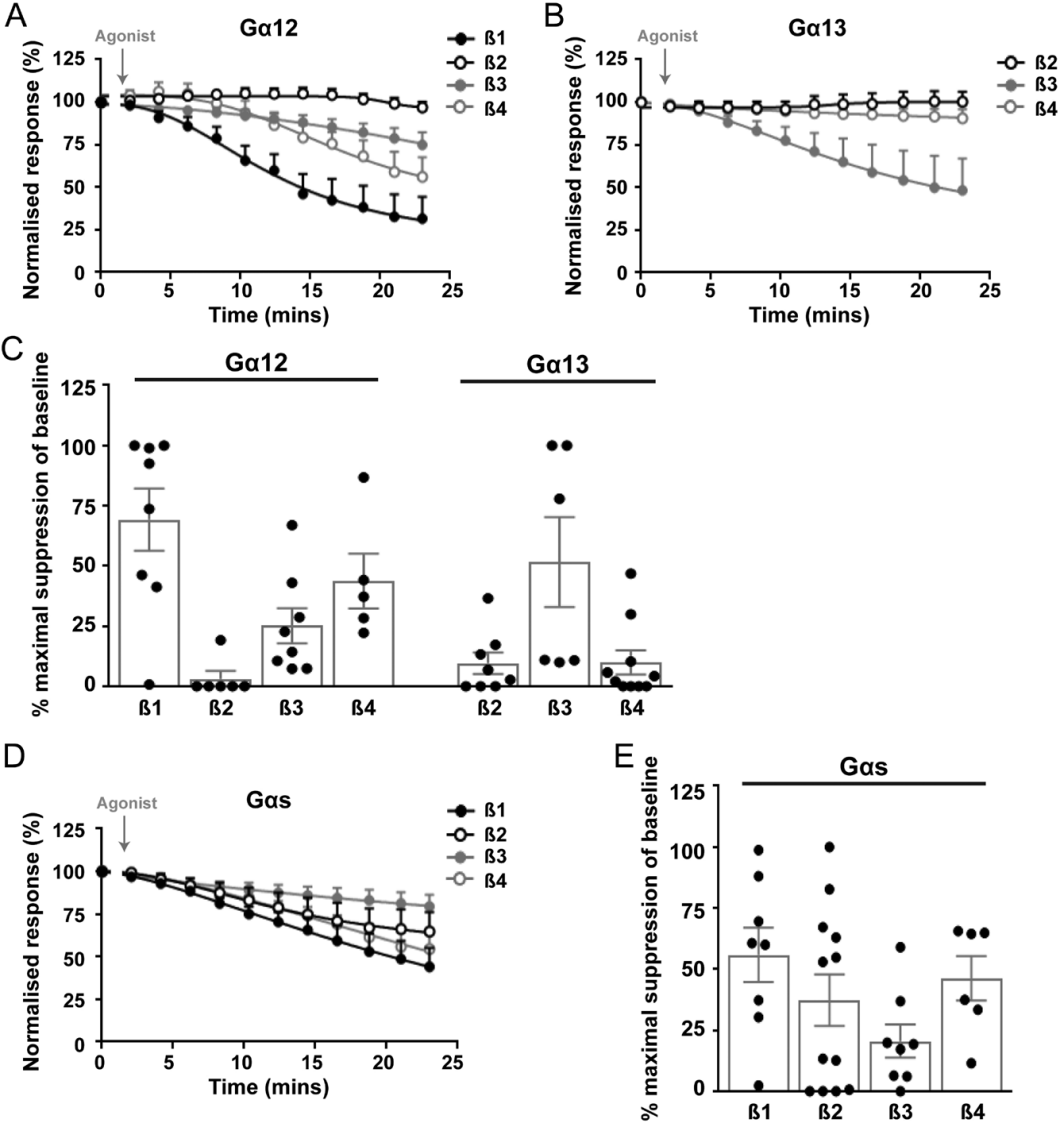
CaSR activation of the G_12/13_ family by NanoBiT G protein dissociation assays. NanoBiT dissociation assays of AdHEK-CaSR cells transiently transfected with: (A) LgBiT-Gα_12_ and (B) LgBiT-Gα_13_ with SmBiT-Gβ subunits (Gβ1–5) and unlabelled Gγ2 following treatment with 5 mM Ca^2+^_e_. Pre-calcium baselines are not shown. The first baseline value is shown. (C) Percentage suppression of baseline from A and B. (D) NanoBiT dissociation assays of AdHEK-CaSR cells transiently transfected with: LgBiT-Gα_s_, SmBiT-Gβ subunits (Gβ1–5) and unlabelled Gγ2 following treatment with 5 mM Ca^2+^_e_. Pre-calcium baselines are not shown. The first baseline value is shown. (E) Percentage suppression of baseline from D. Statistical analyses comparing values of all Gα-Gβ dissociations are shown in Supplementary Table 1. For all panels only those Gα and Gβ pairs that yielded relative luminescence units (RLU) over a threshold of 1 × 10^3^ (i.e. above background luminescence) are shown. All responses were normalised to those under basal conditions (0.1 mM Ca^2+^_e_). Dissociation curves for 5 mM Ca^2+^_e_ are shown (curves with 0.1 mM Ca^2+^_e_ are shown in Supplementary Figs 10 and 11). Curves show mean ± s.e.m. for *n* = 6–12 independent assays.

CaSR has been reported to activate G_12/13_ inducing cytoskeletal changes in some cells, while other studies were unable to show any signalling, indicating the pathway may be cell type specific (Pi *et al.* 2002, Huang *et al.* 2004, Davies *et al.* 2006). NanoBiT dissociation assays in AdHEK-CaSR cells showed Gα_12_ was activated by CaSR when combined with three β-subunits, β1, β2 and β3 (Fig. 3 and Supplementary Fig. 10). However, activation of Gα_12_ with Gβ2 and Gβ3 took more than 12 min, indicating it is unlikely rapid CaSR activation is induced by coupling between these subunits. Similarly, Gα_13_ could only be activated when combined with Gβ4. Thus, activation of Gα_12/13_ by CaSR may only occur when specific β-subunits are present in cells and may explain some of the discrepancies observed between published studies investigating CaSR signalling by this pathway.

Previous studies of CaSR in cancer cell-lines, including MCF-7 human breast cancer cells and mouse pituitary AtT20 cells, have shown that CaSR switches from preferentially coupling to G_q__/11_ and G_i__/o_ pathways to exclusively signalling by a G_s_ pathway (Mamillapalli *et al.* 2008, Mamillapalli & Wysolmerski 2010). Combinations of Gα_s_ with Gβ1–4 achieved luminescence values above the threshold, indicating efficient activation. However, agonist-induced responses were only significantly greater than basal responses from 12 min after stimulations in cells transfected with Gβ1 and after 15 min of stimulation in cells expressing Gβ2 and Gβ4 (Fig. 3 and Supplementary Fig. 11). Therefore, CaSR may activate Gα_s_ and Gα_12/13_ more slowly than other Gα proteins, and this could explain why *in vitro* assays often fail to detect CaSR activation of these pathways.

### Gα_11_, Gα_i1_ and Gα_z_ are most abundantly expressed in human parathyroid tissue

Our NanoBiT dissociation studies have demonstrated which G proteins are capable of coupling to CaSR. However, CaSR coupling in native human tissue will be dependent on the expression of the individual G proteins in that tissue. For example, previous studies using qRT-PCR have indicated that the gene encoding Gα_11_ (*GNA11*) is expressed at higher levels than that of Gα_q_ (*GNAQ*) (Nesbit *et al.* 2013*a*); while in bovine parathyroid, genes for Gα_11_, Gα_s_, Gα_i2_, Gα_12_ and Gα_z_ were highly expressed (Varrault *et al.* 1995). We sought to verify these findings in a larger dataset and investigate G protein expression in the three tissues in which CaSR is most abundantly expressed: the parathyroid glands, the kidneys and the pancreas.

**Figure 4:**
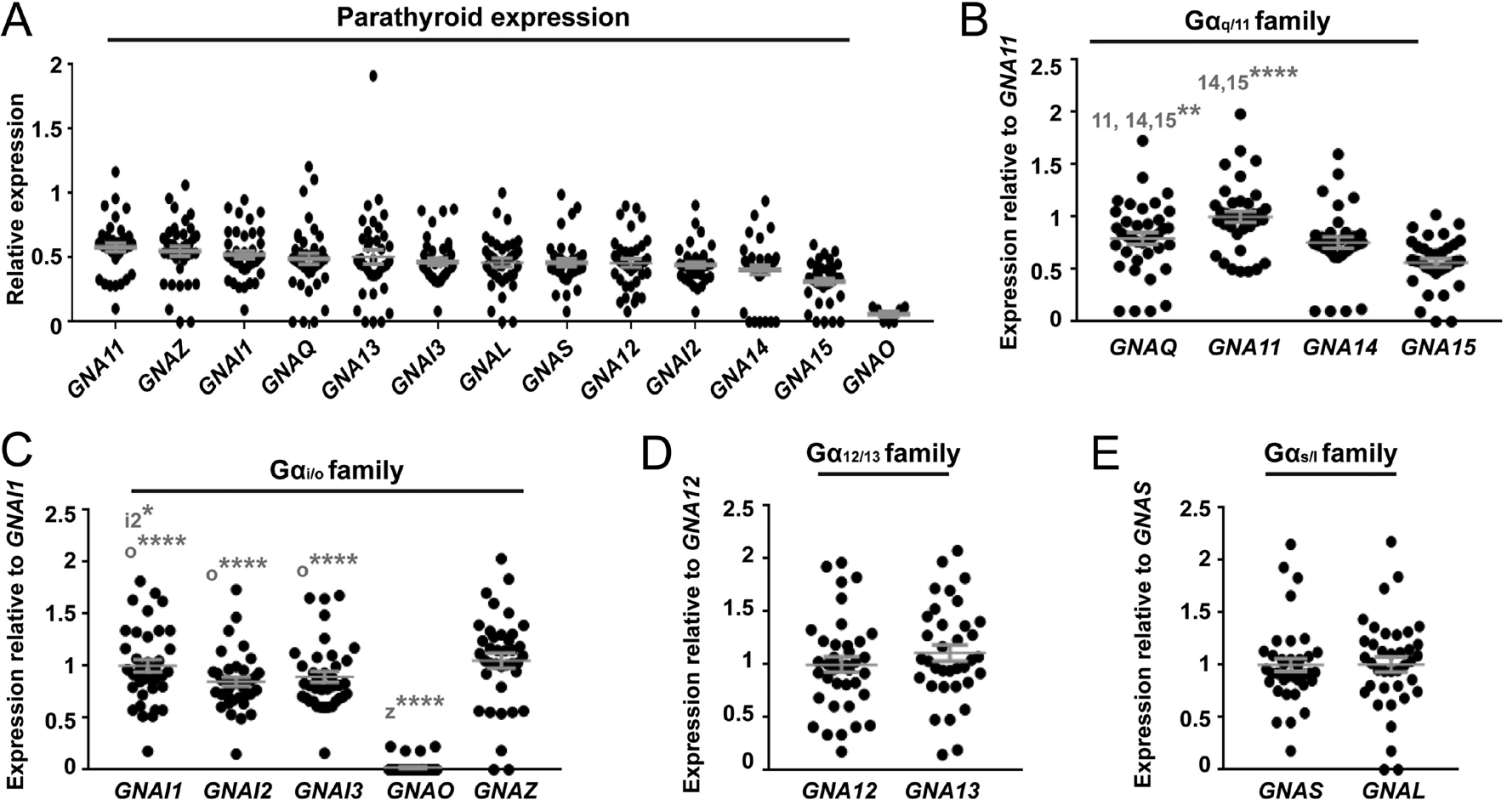
Expression of G protein α-subunits in human parathyroid tissue. (A) The mRNA expression of 13 Gα-subunits in human parathyroid tissue shown as a relative fold-change compared to the geometric mean of control genes. Statistical analyses comparing expression between subunits is shown in Supplementary Table 1. (B, C, D and E) To compare expression of genes within subfamilies, each gene was expressed as a fold-change compared to either: (B) *GNA11*; (C) *GNAI1*; (D) *GNA12;* and (E) *GNAS*. Statistical analyses for panels B, C, D, E and F were performed by one-way ANOVA with multiple comparisons and subunits with which comparisons are made are indicated in grey. ****P < 0.0001, ***P < 0.001, **P < 0.01, *P < 0.05.

**Figure 5:**
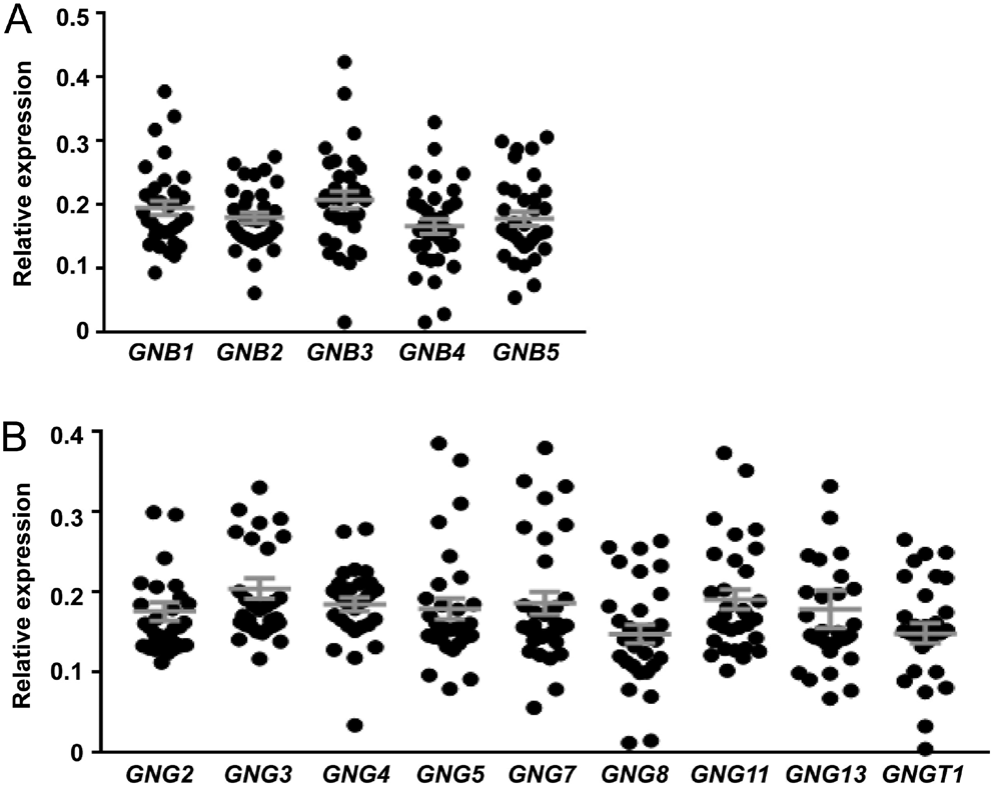
Expression of G protein β-subunits in human parathyroid tissue. The mRNA expression of (A) all five Gβ-subunits and (B) nine Gγ-subunits in human parathyroid tissue shown as a relative fold-change compared to the geometric mean of control genes. Statistical analyses were performed by one-way ANOVA and subunits with which comparisons are made are indicated in grey. **P < 0.01, *P < 0.05.

We first searched the Gene Expression Omnibus (GEO) repository to identify existing gene expression datasets from human parathyroid tissue. This identified: one dataset from expression profiling by array of normal parathyroid glands (*n* = 6) and adenoma tissue from patients with primary hyperparathyroidism (*n* = 25) (GEO accession #GSE83421) (Balenga *et al.* 2017); and one dataset from expression profiling of normal human tissues (*n* = 3 parathyroid tissues) (GEO accession #GSE2193) (Shyamsundar *et al.* 2005). The expression of G protein α, β or γ-subunits was normalised to a panel of housekeeping genes. As no differences were detected between expression levels of any of these genes in the normal vs adenoma tissues, all data were combined. This demonstrated 13 Gα-subunits, five Gβ-subunits and 9 Gγ-subunits were expressed in the majority of parathyroid tissues (Figs 4 and 5). The expression of all Gα subunits was compared in each sample by expression as a ratio of abundance. No Gβ- or Gγ-subunits were consistently overexpressed (Fig. 5). However, these studies confirmed that *GNA11* has greater expression than *GNAQ* (Fig. 4A, B and Supplementary Table 3). Additionally, three other G protein subunits (*GNAZ, GNAI1* and *GNA13*) had comparable expression to *GNA11* (Fig. 4 and Supplementary Table 3). As the NanoBiT dissociation assays demonstrated no or very little coupling to *GNAZ* and *GNA13* (Figs 2 and 3), it is unlikely that CaSR signals via these G proteins in human parathyroid glands; however, this remains to be verified in parathyroid cells. Thus, the higher expression of genes encoding Gα_11_ and Gα_i1_ may, in part, explain the propensity for CaSR to activate Ca^2+^_i_ and reduce cAMP signalling pathways, and the inability of other Gα_q/11_ family members to fully compensate for mutant Gα_11_ in FHH2 and ADH2 patients.

### Gα_s_ is the most abundantly expressed G protein in human kidney and pancreatic tissue

**Figure 6:**
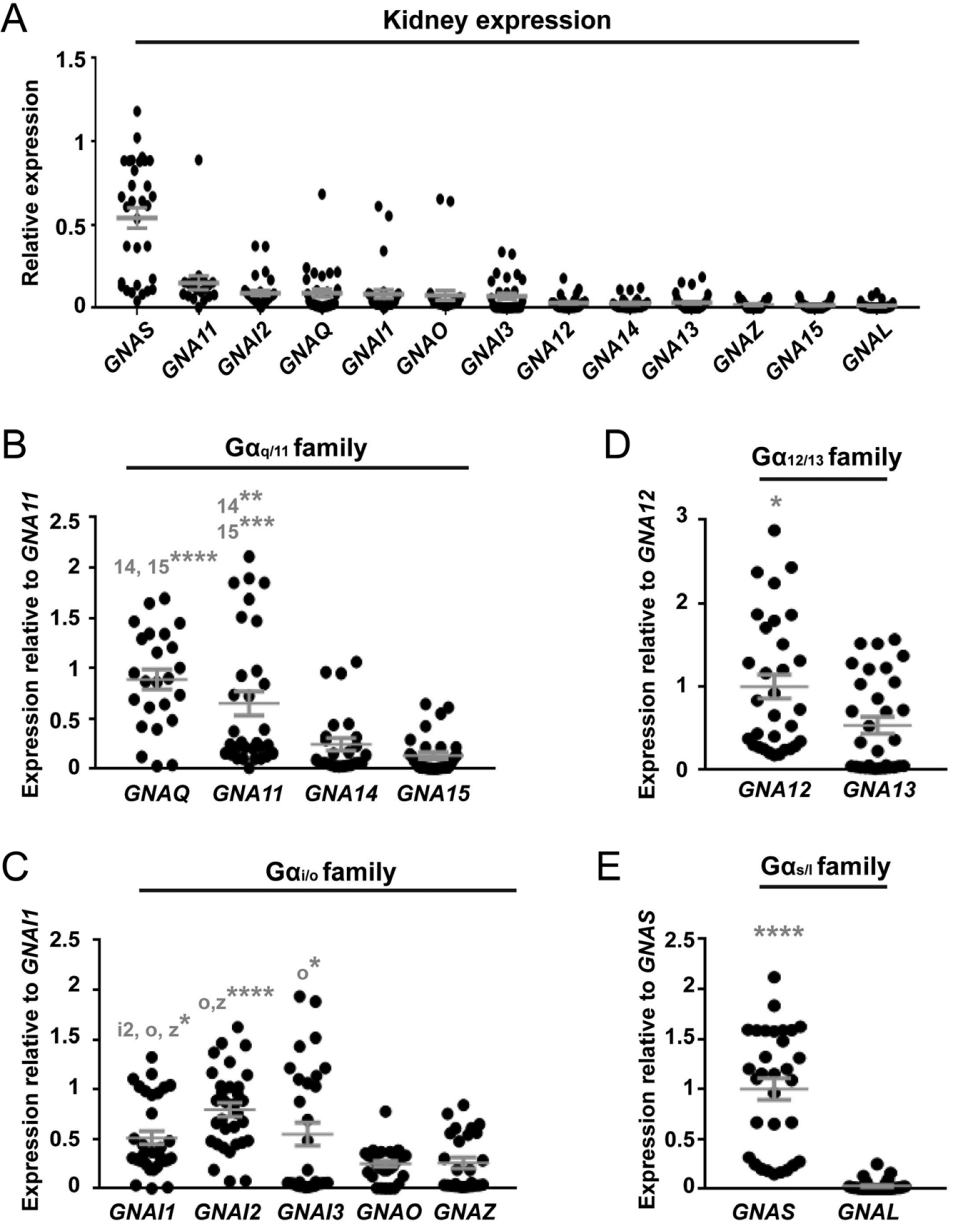
Expression of G protein α-subunits in human kidney tissue. (A) The mRNA expression of 13 Gα-subunits in human kidney tissue shown as a relative fold-change compared to the geometric mean of control genes. Statistical analyses comparing expression between subunits is shown in Supplementary Table 2. (B, C, D and E) To compare expression of genes within subfamilies, each gene was expressed as a fold-change compared to either: (B) *GNA11*; (C) *GNAI1*; (D) *GNA12*; and (E) *GNAS*. Statistical analyses were performed by one-way ANOVA and subunits with which comparisons are made are indicated in grey. ****P < 0.0001, ***P < 0.001, **P < 0.01, *P < 0.05.

Similar analyses were conducted investigating G protein expression in human kidney tissue in the GEO repository. Only datasets investigating expression in non-diseased tissue were included in analyses. Whole kidney samples were used, as datasets for individual kidney cell types were not available. *GNAS* was the most abundantly expressed G protein in kidney tissue (Fig. 6 and Supplementary Table 4). When the data were reanalysed to focus on individual subfamily members, *GNAQ* and *GNA11* were similarly expressed, and both were more abundant than *GNA14* and *GNA15* (Fig. 6B). The *GNAL*, *GNAO* and *GNAZ* genes were poorly expressed, and *GNA12* was expressed at significantly greater levels than *GNA13* (Fig. 6C and D). Thus, within the kidney, CaSR may be able to signal by Gα_q_ or Gα_11_, and may explain why patients with FHH2 and ADH2 mutations have milder symptoms than patients with CaSR mutations in FHH1 or ADH1 (Nesbit *et al.* 2013*a*, Gorvin *et al.* 2018*a*). Additionally, CaSR may signal by several other G proteins that are similarly expressed in kidney tissue.

**Figure 7:**
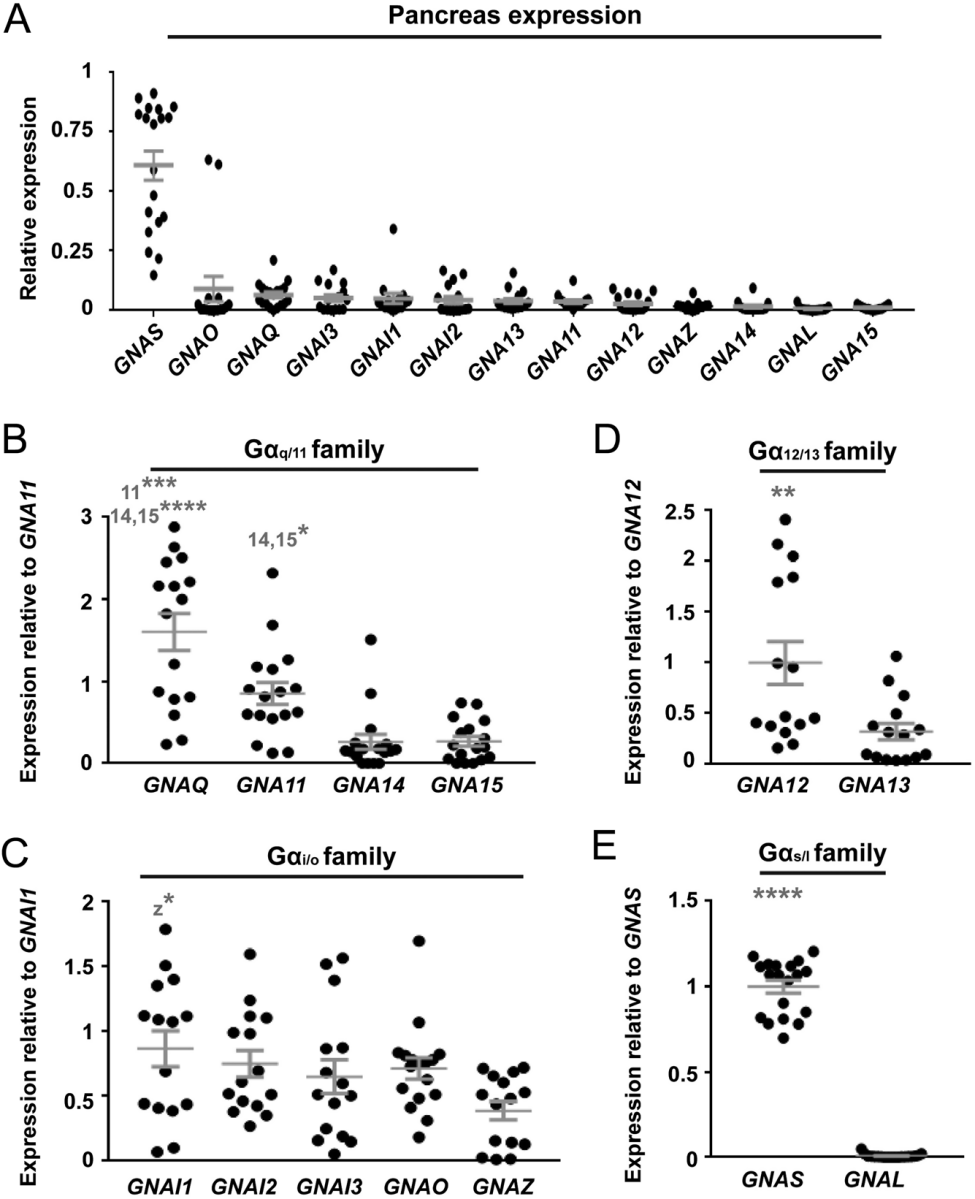
Expression of G protein α-subunits in human pancreatic tissue. (A) The mRNA expression of 13 Gα-subunits in human pancreatic tissue shown as a relative fold-change compared to the geometric mean of control genes. Statistical analyses comparing expression between subunits is shown in Supplementary Table 3. (B, C, D and E) To compare expression of genes within subfamilies, each gene was expressed as a fold-change compared to either: (B) *GNA11*; (C) *GNAI1*; (D) *GNA12*; and (E) *GNAS*. Statistical analyses were performed by one-way ANOVA and subunits with which comparisons are made are indicated in grey. ****P < 0.0001, ***P < 0.001, *P < 0.05.

The CaSR is highly expressed in pancreatic tissue, and roles have been described in α- and β-cells (Squires *et al.* 2000, Regard *et al.* 2008). A number of gene expression datasets are available in the GEO repository, including from the whole pancreas and individual cell types. We chose to analyse whole pancreas samples rather than individual cell types as: CaSR is expressed in multiple cell types; these samples were collected from non-disease tissue; and there were enough samples to perform statistical analyses on. These analyses demonstrated that *GNAS* is the most highly expressed G protein in pancreatic tissue (Fig. 7), while members of the G_i__/o_ and G_q__/11_ families were expressed at similar levels (Fig. 7 and Supplementary Table 5). Analyses within subfamilies demonstrated *GNAQ* and *GNA11* to be more abundantly expressed than *GNA14* and *GNA15*, with *GNAQ* most highly expressed (Fig. 7B). Few differences were observed between subfamily members in the G_i__/o_ family, while Gα_12_ was more highly expressed than *GNA13* (Fig. 7). Therefore, CaSR could couple to many G proteins within pancreatic tissue, and this may explain why no overt pancreatic phenotypes (e.g. diabetes, variations in blood glucose or insulin levels) have been reported in patients with *GNA11* mutations.

## Discussion

Our studies confirm that the CaSR can activate multiple G proteins, and this may account for the increasingly varied functions that have been described for the receptor in a wide range of tissues (Hofer *et al.* 2000, Rossol *et al.* 2012, Yarova *et al.* 2015, Zietek & Daniel 2015). These studies indicate that GPCR coupling in different tissues is most likely governed by the ability of the receptor to activate an individual G protein, as well as the expression of G proteins in different tissues. Thus, in parathyroid tissue, in which CaSR has its predominant role, *GNA11* and *GNAI1* are two of the most abundantly expressed G proteins, and have amongst the highest G protein activity, consistent with previous studies that indicate that parathyroid cells typically activate G_q__/11_ and G_i__/o_ pathways (Ca^2+^_i_ mobilisation and reductions in cAMP, respectively) (Kifor *et al.* 1997, Brown & MacLeod 2001, Hofer & Brown 2003). Other G proteins that are highly expressed in parathyroid tissue, such as Gα_z_, were not activated by CaSR in the NanoBiT dissociation assays, indicating it is unlikely to be important in calcium-mediated responses. These findings would need to be replicated in parathyroid cells to determine whether Gα_z_ is activated by CaSR in this cell type.

These studies showed that CaSR activation of some members of the G_i__/o_ and G_q__/11_ subfamilies is comparable, indicating that these pathways may have similar importance in CaSR-mediated parathyroid activities, as indicated by early studies of PTH secretion (Conigrave 2016). Despite this, many studies of CaSR variants assess only changes in Ca^2+^_i_, and thus disease-causing mutations in CaSR could be overlooked by these approaches. It is likely researchers have focussed on Ca^2+^_i_ pathways rather than cAMP as fewer robust assays to assess cAMP were available until recent years. Newer assays, such as the cAMP Glosensor system, which allows cAMP to be assessed kinetically, have been increasingly used to assess cAMP signalling by GPCRs (Kumar *et al.* 2017, Lotta *et al.* 2019). However, these assays often still require forskolin treatment to elevate intracellular cAMP levels, and thus increase assay-to-assay variability. Therefore, the development of the NanoBiT dissociation assay will allow both G_q__/11_ and G_i__/o_ pathways to be compared in cells expressing CaSR variant proteins without the need to pre-treat cells with forskolin.

Our studies showed that CaSR could couple to Gα_12_ and Gα_13_, as indicated by previous studies (Huang *et al.* 2004), but that rates of activation were slower than with other G protein families. Slow rates of nucleotide exchange and GTP hydrolysis for G_12/13_ have been described for other GPCRs, and it has been suggested that this may facilitate temporal control over cell processes that require prolonged signalling (Siehler 2009). These slower activation rates could explain why some laboratories have been unable to detect CaSR-mediated G_12/13_ signalling. Alternatively, G_12/13_ signalling may not have been detected in previous studies, as CaSR appears to activate only select combinations of G_12/13_ and Gβ-subunits (Fig. 3), despite coupling between Gα_12/13_ and Gβ1-4 (Supplementary Table 1). Post-translational modifications, including palmitoylation and myristoylation, can affect G-protein recruitment and activation (Bhattacharyya & Wedegaertner 2000), and this may have affected the ability of Gα_12/13_ to activate some Gβ subunits. Further studies investigating the effects of lipid modifications on CaSR signalling by G_12/13_ would be required to determine whether this could explain differences between activation within members of the same subfamilies.

Previous studies have suggested that patients with FHH2 due to Gα_11_ mutations may have a milder phenotype than patients with CaSR mutations, as other G proteins could compensate for Gα_11_ functions (Nesbit *et al.* 2013*a*, Gorvin *et al.* 2018*a*). Our studies demonstrate that CaSR can activate all members of the G_q__/11_ subfamily, and therefore, in principle, these proteins could compensate for impaired Gα_11_ functionality. However, consistent with previous studies, *GNA11* was expressed at significantly greater levels than *GNAQ* in human parathyroid tissue, which may explain why Gα_q_ cannot entirely compensate for Gα_11_, and patients with inactivating Gα_11_ mutations develop mild hypercalcaemia (Nesbit *et al.* 2013*a*). In contrast to parathyroid tissue, expression of *GNA11* and *GNAQ* are not significantly different in kidney tissue, thus compensation by other subunits may be present in kidney cells, providing a reason why phenotypes may be milder than that observed in FHH1 and ADH1. Further investigation of CaSR coupling to mutant Gα_11_ in cells depleted of other G_q__/11_ subfamily members may be required to investigate Gα-protein compensation in greater detail.

In pancreatic tissue, expression of Gα-subunits of the G_i__/o_ family were not significantly different to some members of the G_q__/11_ family. The NanoBiT dissociation assays demonstrated that CaSR robustly activates Gα_i1_ and Gα_o_, both of which are expressed at comparable levels in the pancreas. Thus, CaSR-mediated functions in pancreatic cells may arise by coupling to either G protein family. Activation of the G_i__/o_ family that negatively modulates cAMP signalling and is important in reducing hormone secretion by other GPCRs (Braun 2014), could explain how CaSR activation impairs hormone secretion in pancreatic cells. However, previous studies in pancreatic β-cells failed to show a role for CaSR-mediated cAMP depletion in insulin secretion (Squires *et al.* 2000), and therefore further studies of CaSR coupling in pancreatic β-cells may be required.

Studies of GPCR signalling using a NanoBiT G protein dissociation assay system has several advantages over other methodologies to study GPCR activation. The ability to assess multiple G protein pathways in parallel reduces the need to perform different assays to assess individual signalling pathways, and avoids the need to chemically or genetically inhibit gene expression, allowing the researcher to directly compare responses by each G protein. This will revolutionise the ability to assess biased signalling by disease-causing CaSR mutations which has been described in several studies (Leach *et al.* 2012, Gorvin *et al.* 2018*b*,*c*) and will simplify comparisons between CaSR allosteric modulators. Furthermore, the simplicity of the methodology and availability of these plasmids will allow researchers in different laboratories to directly compare results, increasing the robustness and reproducibility of data yielded from these assays. The ability to collect kinetic data easily, which many assays that have been used previously to measure GPCR signalling cannot do (e.g. IP-one, LANCE cAMP, pERK AlphaScreen, luciferase reporter genes), will increase our understanding of GPCR temporal signalling. This was demonstrated for the CaSR as our NanoBiT assays showed activation of Gα_12/13_ and Gα_s_ occurs more slowly than activation of other Gα-proteins. If this assay system is to be adopted by multiple laboratories, it will be important that a uniform methodology is used. For example, the time between agonist addition and measurement of luminescence should always be the same in all laboratories, to allow rates of dissociation to be compared.

A disadvantage of this study is that it was conducted in a single cell-line, adherent HEK293 overexpressing CaSR, which could be criticised as an artificial system. We chose this cell-line as HEK293: are easy to grow and transfect; are available to most research labs so findings can be replicated; are routinely used in CaSR research; and cultures of parathyroid and kidney cell-lines are not widely available. Moreover, the purpose of this study was to determine whether CaSR can couple to all G protein subunits and establish a simple assay system to assess CaSR variants and potentially screen pharmacological compounds to determine their ability to engender signalling bias. Future studies investigating endogenous G protein activation by CaSR in specific cell types may become possible with recently published BRET constructs (Maziarz *et al.* 2020). However, these assays still require transfection and, therefore, may be difficult in primary cells. Our study could also be criticised for only assessing one CaSR ligand. We assessed Ca^2+^_e_ as it is the best characterised ligand in *in vitro* assays of CaSR signalling and is the major physiological agonist of the receptor. Other studies have described CaSR activation by other ligands, including aromatic amino acids and polyamines (Conigrave *et al.* 2000, 2004, Thomsen *et al.* 2012). However, many of these act as modulators of Ca^2+^_e_-mediated responses, or physiological functions are unknown. Future studies could utilise the NanoBiT dissociation assay system to compare the effects of these ligands by a single methodology. Finally, the NanoBiT assay we describe will be unable to detect G protein activation in which Gα-Gβγ dissociation does not occur. Studies have indicated that such activation in the absence of Gα-Gβγ dissociation may occur in some cell types (e.g. cone photoreceptors (Rosenzweig *et al.* 2007)). However, the number of GPCRs able to utilise such signalling is unknown, and it is possible that some studies that reported the absence of dissociation may not have measured signal kinetics, and thus may have missed slower dissociation rates that have been reported by some laboratories (Digby *et al.* 2008). The NanoBiT dissociation assay, will be able to detect these differences in dissociation rates as shown for Gα_12/13_ and Gα_s_.

In conclusion, we have demonstrated that CaSR differentially activates multiple Gα proteins. Physiological effects of CaSR are likely mediated by a combination of G protein activation and expression of individual Gα-proteins in different tissues. These studies highlight a simple, single assay system that can be used to robustly assess biased signalling and could be utilised in the development of new pharmacological compounds targeting the CaSR.

## Supplementary Material

Supplementary Figure 1 G protein dissociation curves to determine the optimal concentration of Ca2+e NanoBiT dissociation assays in AdHEK293 transiently transfected with CaSR, LgBiT-Gα proteins, SmBiT-Gβ3 and unlabelled Gγ2. The first four points show responses under baseline conditions, followed by responses after agonist (indicated by a grey arrow). Cells were treated with five concentrations of Ca2+e as indicated above each graph. Gαz is not shown as no dissociation was observed at any concentration of Ca2+e. Subsequent studies to assess all Gα and Gβ combinations were performed at 5mM Ca2+e as the majority of Gα proteins responded to this concentration of calcium. All responses were normalised to those under basal conditions (0.1mM Ca2+e). Curves show mean±SEM for n = 3 independent assays.

Supplementary Figure 2 G protein dissociation curves in AdHEK and AdHEK-CaSR cells NanoBiT dissociation assays in AdHEK cells transiently transfected with LgBiT-Gα proteins, SmBiT-Gβ3 and unlabelled Gγ2 and either pcDNA3.1-FLAG or pcDNA3.1-FLAG-CaSR. Cells were treated with 5mM Ca2+e. All responses were normalised to those under basal conditions (0.1mM Ca2+e). Curves show mean±SEM for n = 3 independent assays.

Supplementary Figure 3 Assessment of the effect of untagged Gα protein overexpression on NanoBiT dissociation (A) NanoBiT dissociation assay of LgBiT-Gαq with SmBiT-Gβ2 in AdHEK cells transiently transfected with either untagged Gα11, Gαi1, Gα12, Gαs. Cells were treated with 5mM Ca2+e. All responses were normalised to those under basal conditions (0.1mM Ca2+e), which are not shown. Curves show mean±SEM for n = 4 independent assays. (B) Quantification of the area between 100% and the maximal inhibitory value (Imax) from each curve in A. Overexpression of untagged Gα proteins had no effect on NanoBiT dissociation.

Supplementary Figure 4 G protein dissociation assessed in G protein knockout cells (A-D) NanoBiT dissociation assays in G protein knockout (KO) cells or parental HEK293 transiently transfected with CaSR, untagged Gγ2 and the LgBiT-Gα and SmBiT-Gβ indicated above each graph, with quantification of the area between 100% and the maximal inhibitory value (Imax) from each curve. Absence of different G proteins had no effect on NanoBiT dissociation assays. Cells were treated with 5mM Ca2+e. All responses were normalised to those under basal conditions (0.1mM Ca2+e), which are not shown. Curves show mean±SEM for n = 3 independent assays.

Supplementary Figure 5 Establishment of an AdHEK cell-line stably overexpressing CaSR (A) Western blot analyses showing overexpression of CaSR in AdHEK-CaSR stable cell-lines and absence of expression in untransfected cells (labelled AdHEK). (B) NanoBiT dissociation curves for LgBiT-Gαq and SmBiT-β3 in AdHEK-CaSR clones A-C, showing similar responses to 5mM Ca2+e. (C) Quantification of the area between 100% and the maximal inhibitory value (Imax) for the responses in B, showing no significant difference between the three clones tested. (D) NanoBiT dissociation curves for Gα11 and β4 in AdHEK-CaSR clones A-C, showing similar responses to 5mM Ca2+e. (E) Quantification of the area between 100% and the maximal inhibitory value for the responses in D, showing no significant difference between the three clones tested. As no significant difference was observed between the three clones subsequent studies were performed in a single clone (clone A). (F) IP3 NanoBiT biosensor assays showing AdHEK-CaSR cells produce IP3 in a dose-dependent manner. (G) cAMP GloSensor measurements of forskolin (Fsk)-generated cAMP production. Exposure of cells to forskolin with 5mM Ca2+e reduces the amount of cAMP generated by AdHEK-CaSR cells. AdHEK cells without CaSR exhibit a forskolin-induced increase in cAMP, similar to AdHEK-CaSR cells, but do not respond to 5mM Ca2+e. Grey arrows on panels B and C show where agonist was added to wells. Data is shown as mean±SEM for n = 4 independent assays.

Supplementary Figure 6 Expression of CaSR and G proteins in cells transfected with NanoBiT constructs Western blot analyses of AdHEK-CaSR cells transfected with LgBiT-Gα proteins, SmBiT-Gβ3 and unlabelled Gγ2. Analyses show transfection of NanoBiT constructs had no effect on: (A) total CaSR protein expression, (B) cell surface expression, measured by assessing CaSR in the plasma membrane fraction, and (C) expression of other G proteins. Two G proteins were tested as examples. Antibodies to G proteins are not specific enough to distinguish between different family members of the same sub-family (e.g. Gαq and Gα11).

Supplementary Figure 7 NanoBiT G-protein dissociation assays of the Gq/11 subfamily NanoBiT dissociation assays of AdHEK-CaSR cells transiently transfected with: LgBiT-Gα (q, 11, 14, 15), SmBiT-Gβ subunits (Gβ 1 - 5) and unlabelled Gγ2. Each panel shows dissociation when cells were exposed to 0.1mM Ca2+e (open, white circle) or 5mM Ca2+e (black, closed circles). Grey arrow indicates when agonist was added. Curves show mean±SEM for n = 6-11 independent assays.

Supplementary Figure 8 NanoBiT G-protein dissociation assays of the Gi/o subfamily NanoBiT dissociation assays of AdHEK-CaSR cells transiently transfected with: LgBiT-Gα (i1, i2, i3, o or z), SmBiT-Gβ subunits (Gβ 1 - 5) and unlabelled Gγ2. Each panel shows dissociation when cells were exposed to 0.1mM Ca2+e (open, white circle) or 5mM Ca2+e (black, closed circles). Grey arrow indicates when agonist was added. Curves show mean±SEM for n = 6-10 independent assays.

Supplementary Figure 9 NanoBiT G-protein dissociation assay showing SSTR5 activates Gαz NanoBiT dissociation assays of AdHEK cells transiently transfected with: pcDNA-SSTR5, LgBiT-Gαz, SmBiT-Gβ4 and unlabelled Gγ2. Cells were exposed to vehicle (DMSO) or 50nM somatostatin (SST). Curves show mean±SEM for n = 4 independent assays.

Supplementary Figure 10 NanoBiT G-protein dissociation assays of the G12/13 subfamily NanoBiT dissociation assays of AdHEK-CaSR cells transiently transfected with: LgBiT-Gα (12 or 13), SmBiT-Gβ subunits (Gβ 1 - 5) and unlabelled Gγ2. Each panel shows dissociation when cells were exposed to 0.1mM Ca2+e (open, white circle) or 5mM Ca2+e (black, closed circles). Grey arrow indicates when agonist was added. Curves show mean±SEM for n = 6-12 independent assays.

Supplementary Figure 11 NanoBiT G-protein dissociation assays of the Gs/l subfamily NanoBiT dissociation assays of AdHEK-CaSR cells transiently transfected with: LgBiT-Gαs, SmBiT-Gβ subunits (Gβ 1 - 5) and unlabelled Gγ2. Each panel shows dissociation when cells were exposed to 0.1mM Ca2+e (open, white circle) or 5mM Ca2+e (black, closed circles). Grey arrow indicates when agonist was added. Curves show mean±SEM for n = 6-12 independent assays.

Supplementary Table 1 Relative luminescence units for Gα-Gβγ pairings used in NanoBiT analyses

Supplementary Table 2 Statistical analyses comparing % maximal suppression of baseline for NanoBiT dissociation assays

Supplementary Table 3 Statistical analyses comparing expression of G-protein α-subunits in human parathyroid tissue

Supplementary Table 4 Statistical analyses comparing expression of G-protein α-subunits in human kidney tissue

Supplementary Table 5 Statistical analyses comparing expression of G-protein α-subunits in human pancreatic tissue

## Declaration of interest

The authors declare that there is no conflict of interest that could be perceived as prejudicing the impartiality of the research reported.

## Funding

This work was funded by: start-up funds to C M G from the Centre of Membrane Proteins and Receptors (COMPARE) and an Academy of Medical Sciences Springboard Award (Ref: SBF004|1034, to C M G), which is supported by the British Heart Foundation, Diabetes UK, the Global Challenges Research Fund, the Government Department of Business, Energy and Industrial Strategy and the Wellcome Trust. A I was funded by PRIME (19gm5910013) and Leading Advanced Projects for Medical Innovation (LEAP) (JP19gm0010004) from the Japan Agency for Medical Research and Development.
