# Supplementary figures and images for: Heterogeneity of G protein activation by the calcium-sensing receptor
Source: J Mol Endocrinol. 2021 Jun 2;67(2):41–53. doi: 10.1530/JME-21-0058 (PMC8240730; doi:10.1530/JME-21-0058)

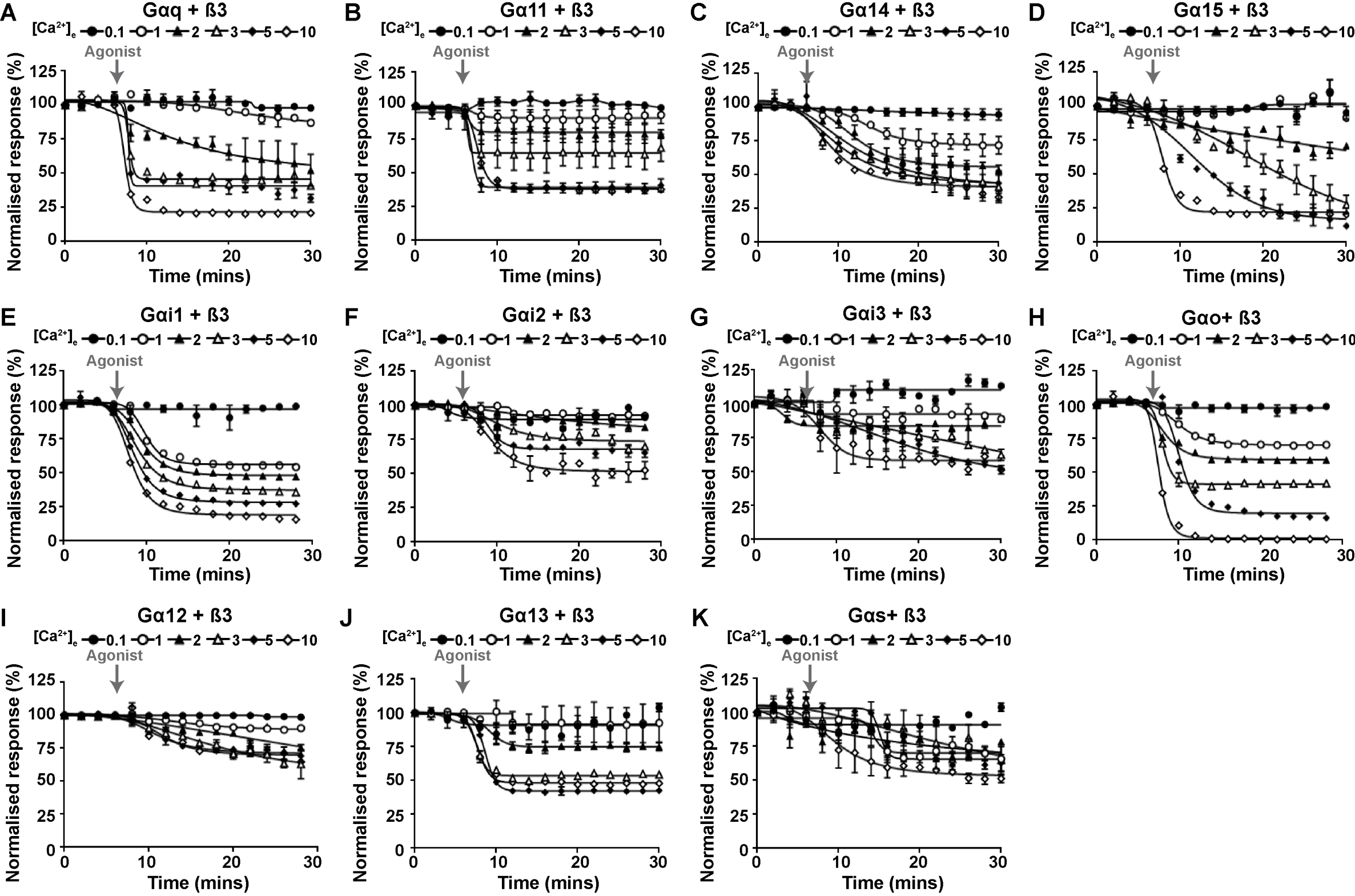

Supplement: Supplementary Figure 1 G protein dissociation curves to determine the optimal concentration of Ca2+e NanoBiT dissociation assays in AdHEK293 transiently transfected with CaSR, LgBiT-Gα proteins, SmBiT-Gβ3 and unlabelled Gγ2. The first four points show responses under baseline conditions, followed by [file supplementary_figure_1.pdf]

**A****Gα11 + β1**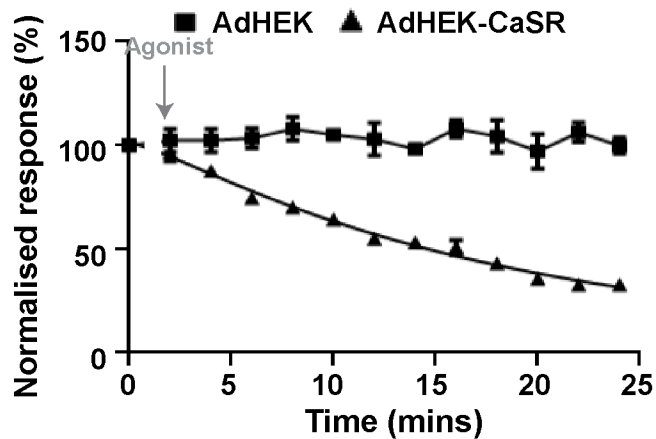**B****Gαi1 + β1**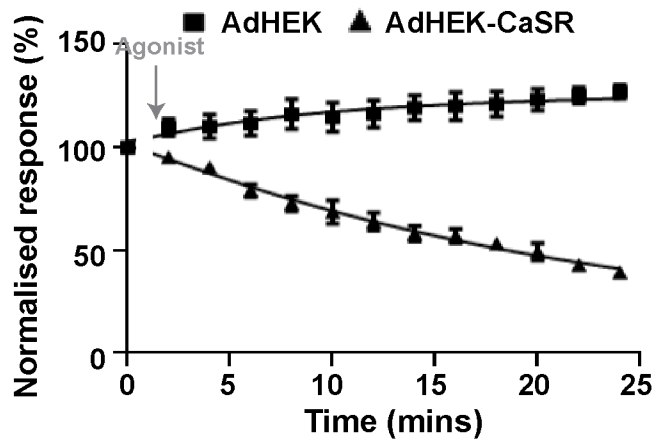**C****Gα12 + β1**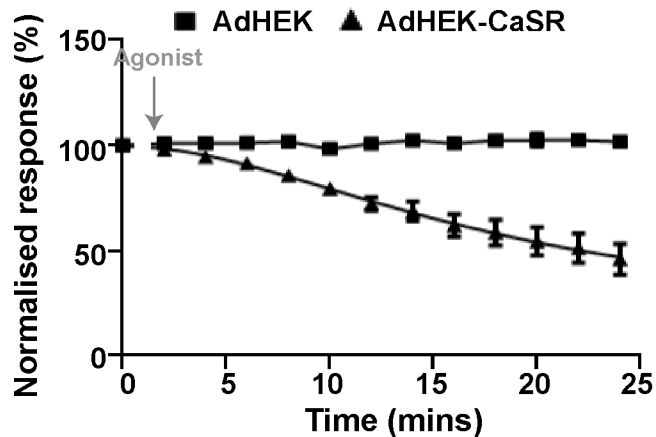**D****Gαs + β1**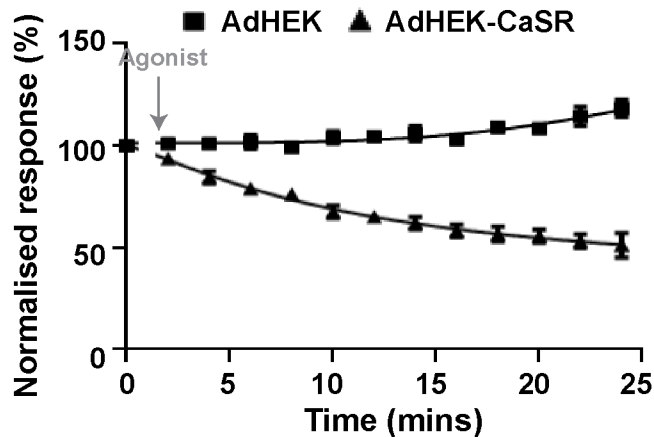

Supplement: Supplementary Figure 2 G protein dissociation curves in AdHEK and AdHEK-CaSR cells NanoBiT dissociation assays in AdHEK cells transiently transfected with LgBiT-Gα proteins, SmBiT-Gβ3 and unlabelled Gγ2 and either pcDNA3.1-FLAG or pcDNA3.1-FLAG-CaSR. Cells were treated with 5mM Ca2+e. All responses  [file supplementary_figure_2.pdf]

**A**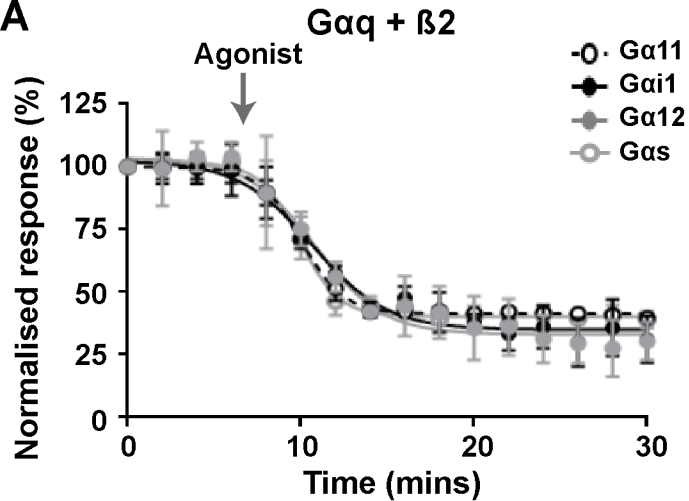**B**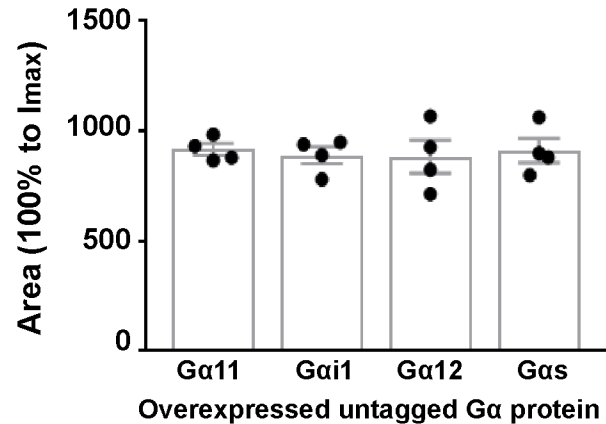

Supplement: Supplementary Figure 3 Assessment of the effect of untagged Gα protein overexpression on NanoBiT dissociation (A) NanoBiT dissociation assay of LgBiT-Gαq with SmBiT-Gβ2 in AdHEK cells transiently transfected with either untagged Gα11, Gαi1, Gα12, Gαs. Cells were treated with 5mM Ca2+e. All responses [file supplementary_figure_3.pdf]

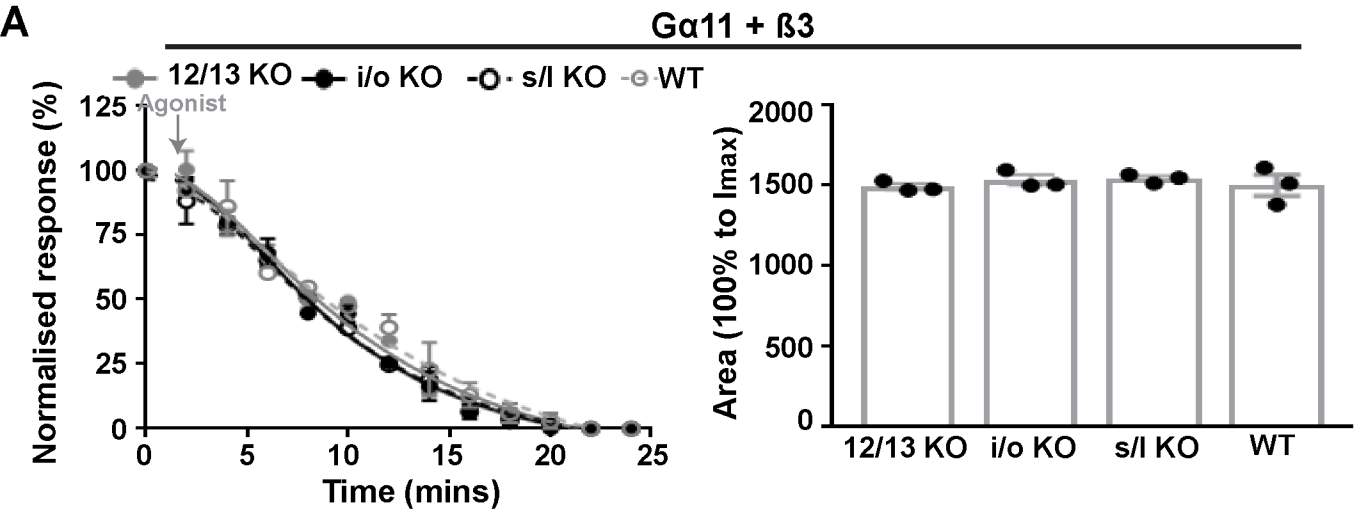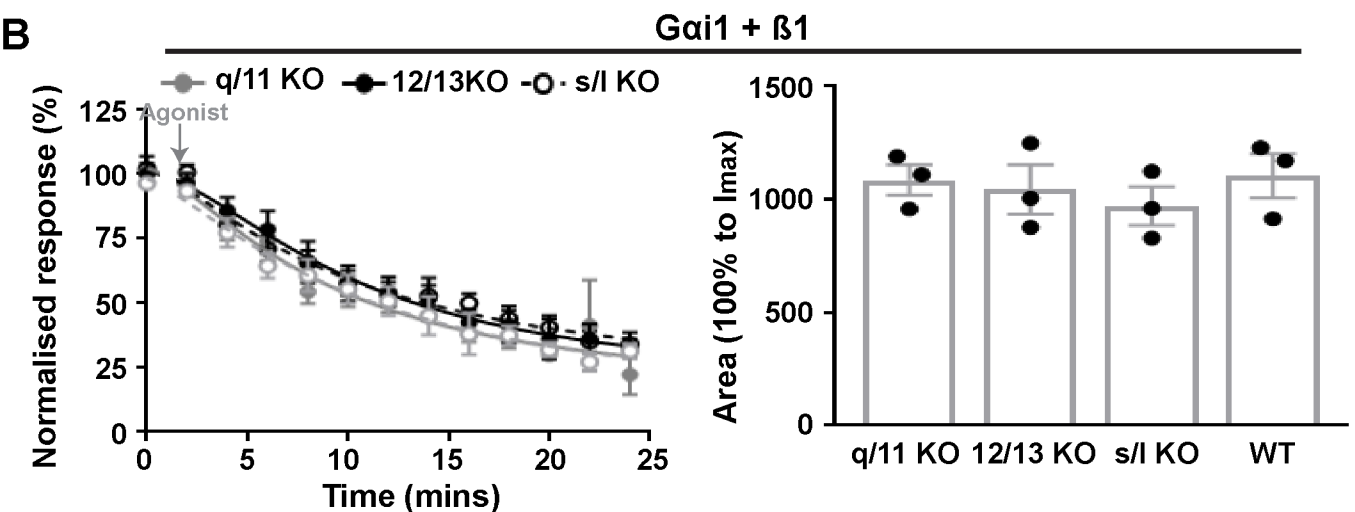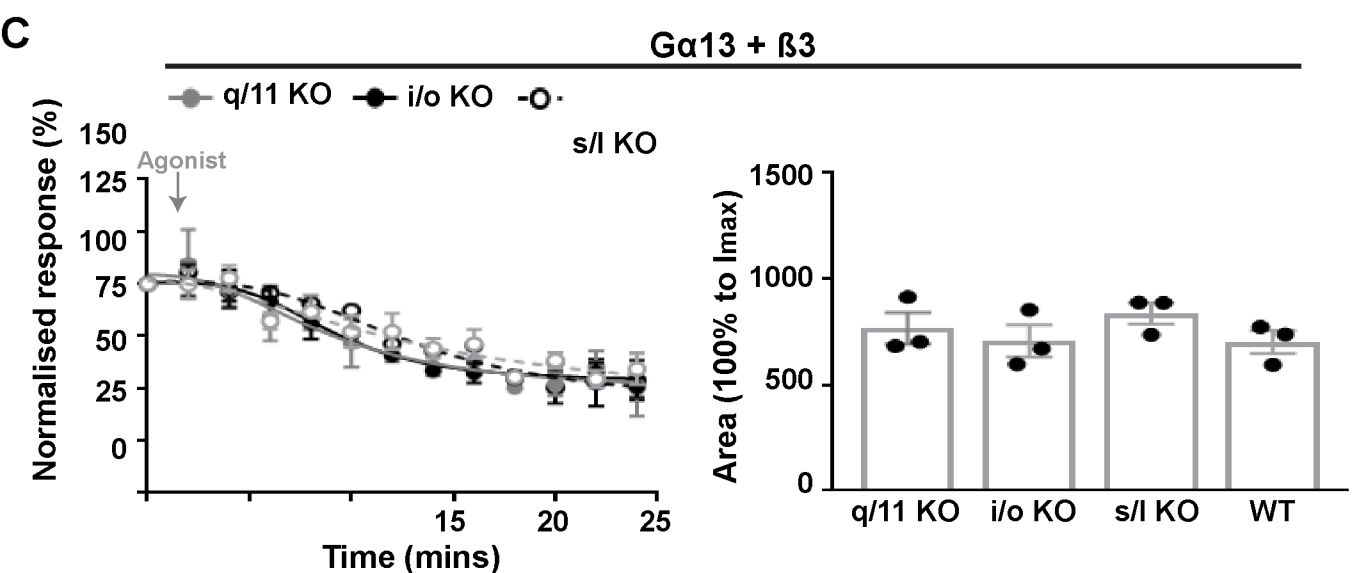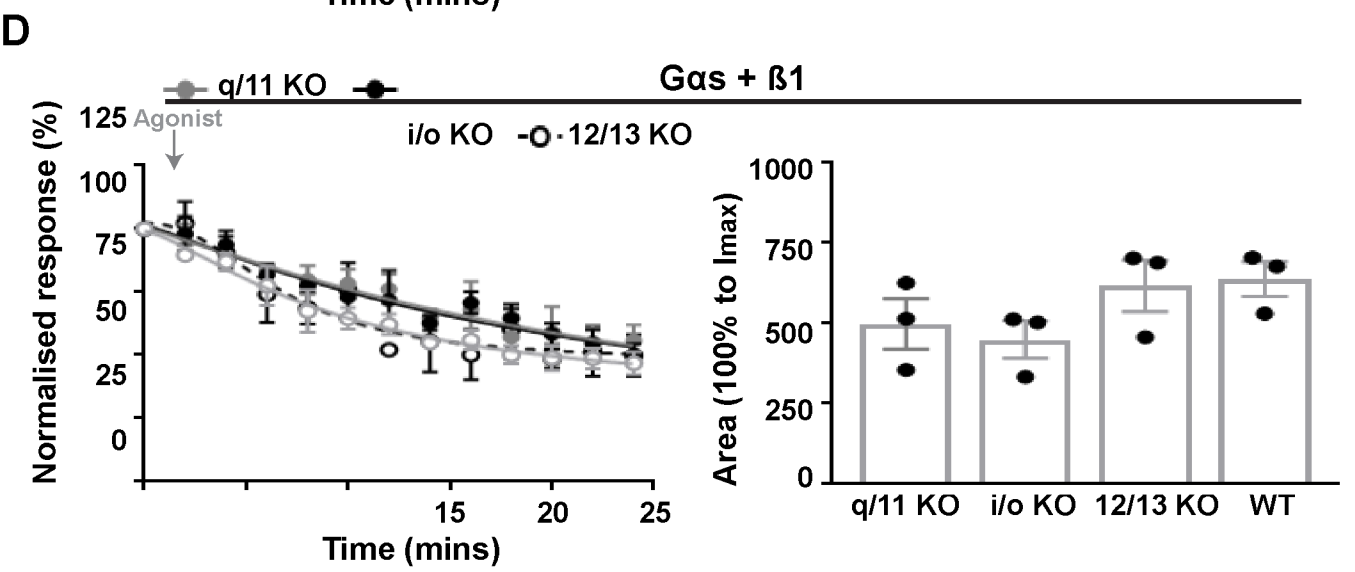

Supplement: Supplementary Figure 4 G protein dissociation assessed in G protein knockout cells (A-D) NanoBiT dissociation assays in G protein knockout (KO) cells or parental HEK293 transiently transfected with CaSR, untagged Gγ2 and the LgBiT-Gα and SmBiT-Gβ indicated above each graph, with quantification of th [file supplementary_figure_4.pdf]

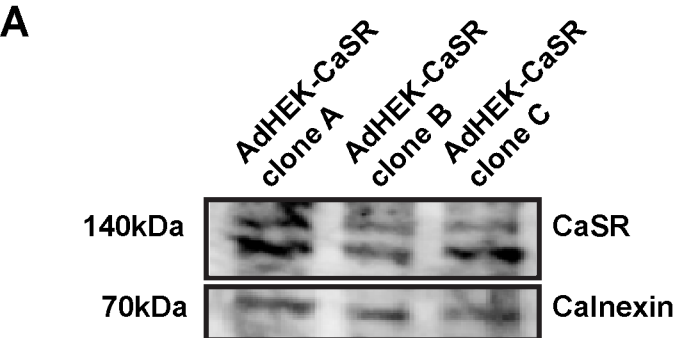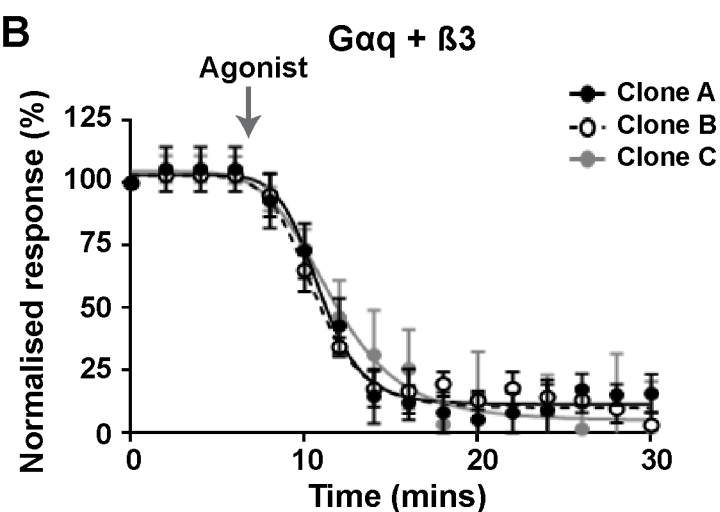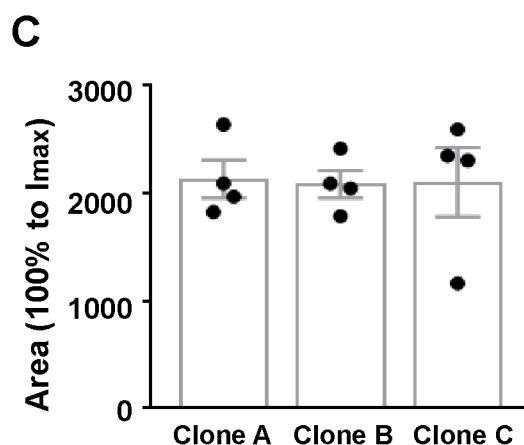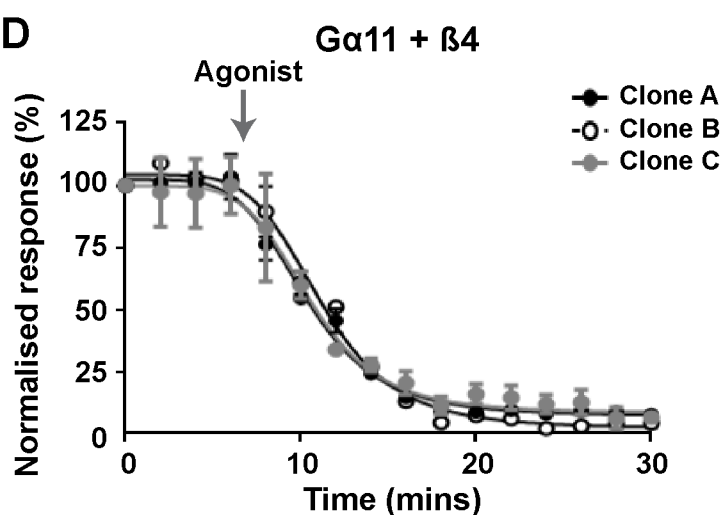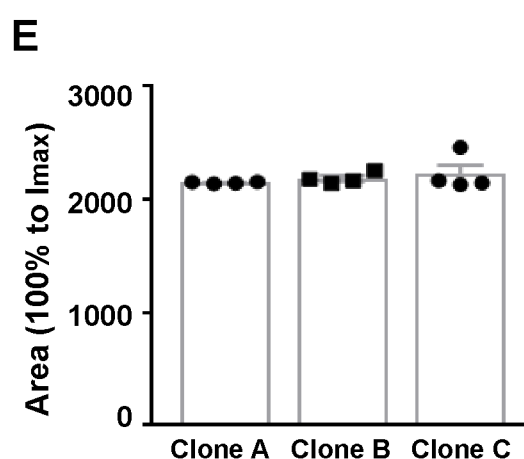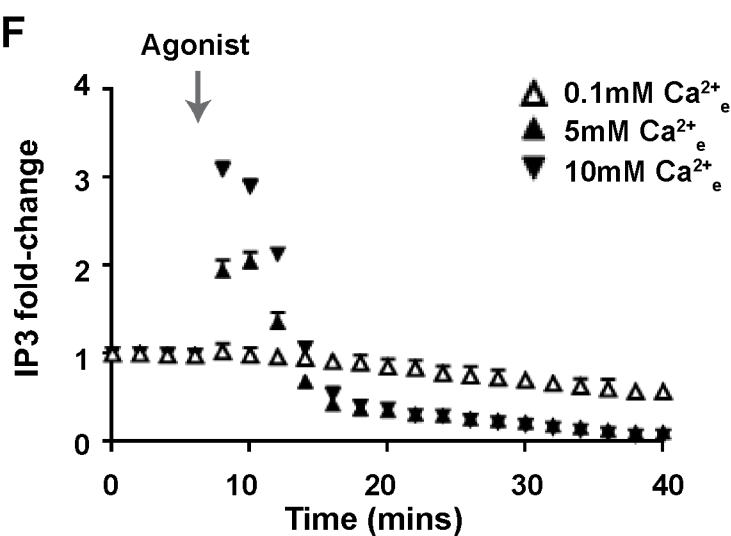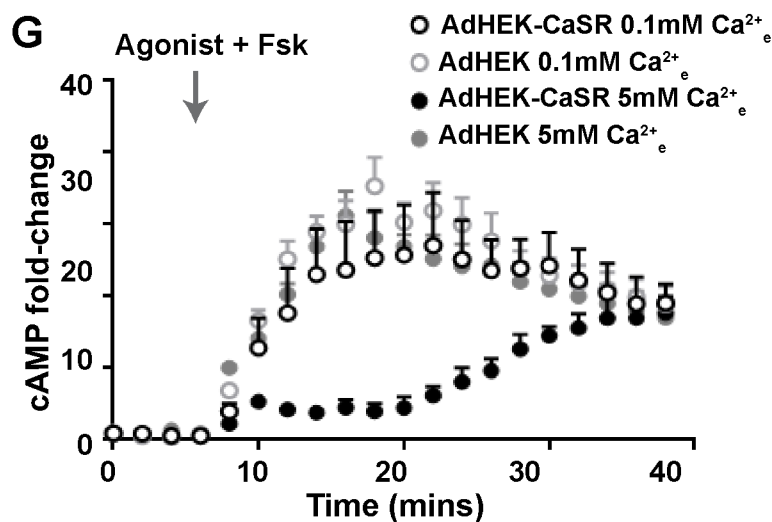

Supplement: Supplementary Figure 5 Establishment of an AdHEK cell-line stably overexpressing CaSR (A) Western blot analyses showing overexpression of CaSR in AdHEK-CaSR stable cell-lines and absence of expression in untransfected cells (labelled AdHEK). (B) NanoBiT dissociation curves for LgBiT-Gαq and SmBiT-β3 [file supplementary_figure_5.pdf]

**A**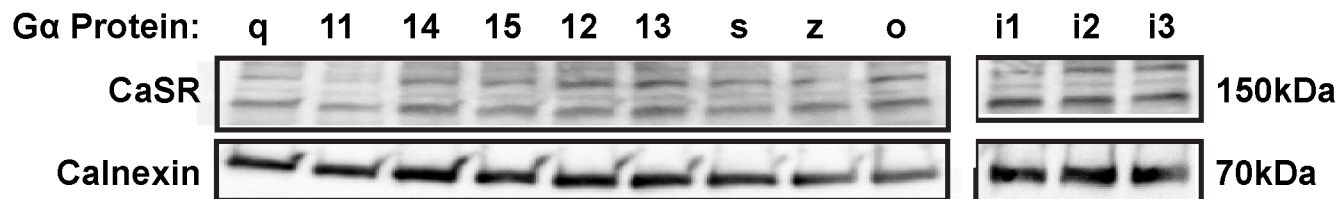**B**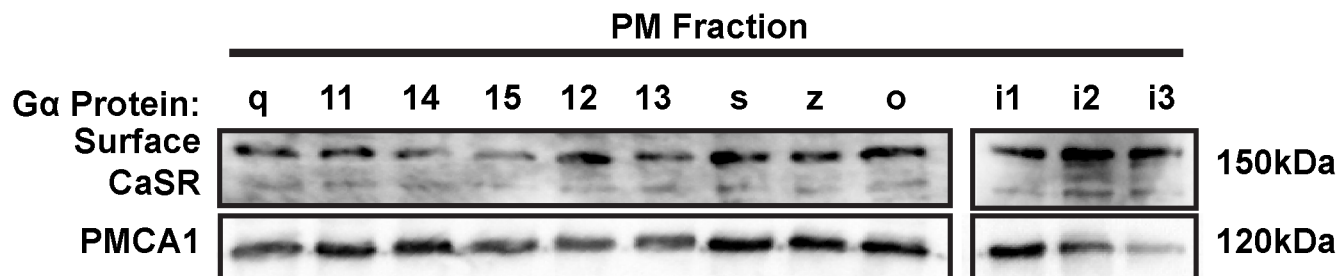**C**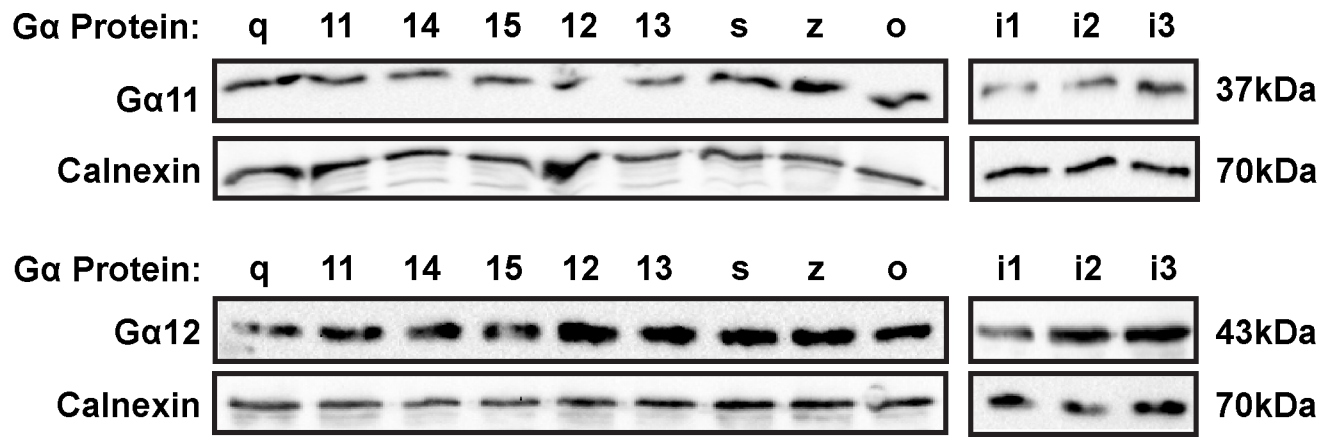

Supplement: Supplementary Figure 6 Expression of CaSR and G proteins in cells transfected with NanoBiT constructs Western blot analyses of AdHEK-CaSR cells transfected with LgBiT-Gα proteins, SmBiT-Gβ3 and unlabelled Gγ2. Analyses show transfection of NanoBiT constructs had no effect on: (A) total CaSR protein  [file supplementary_figure_6.pdf]

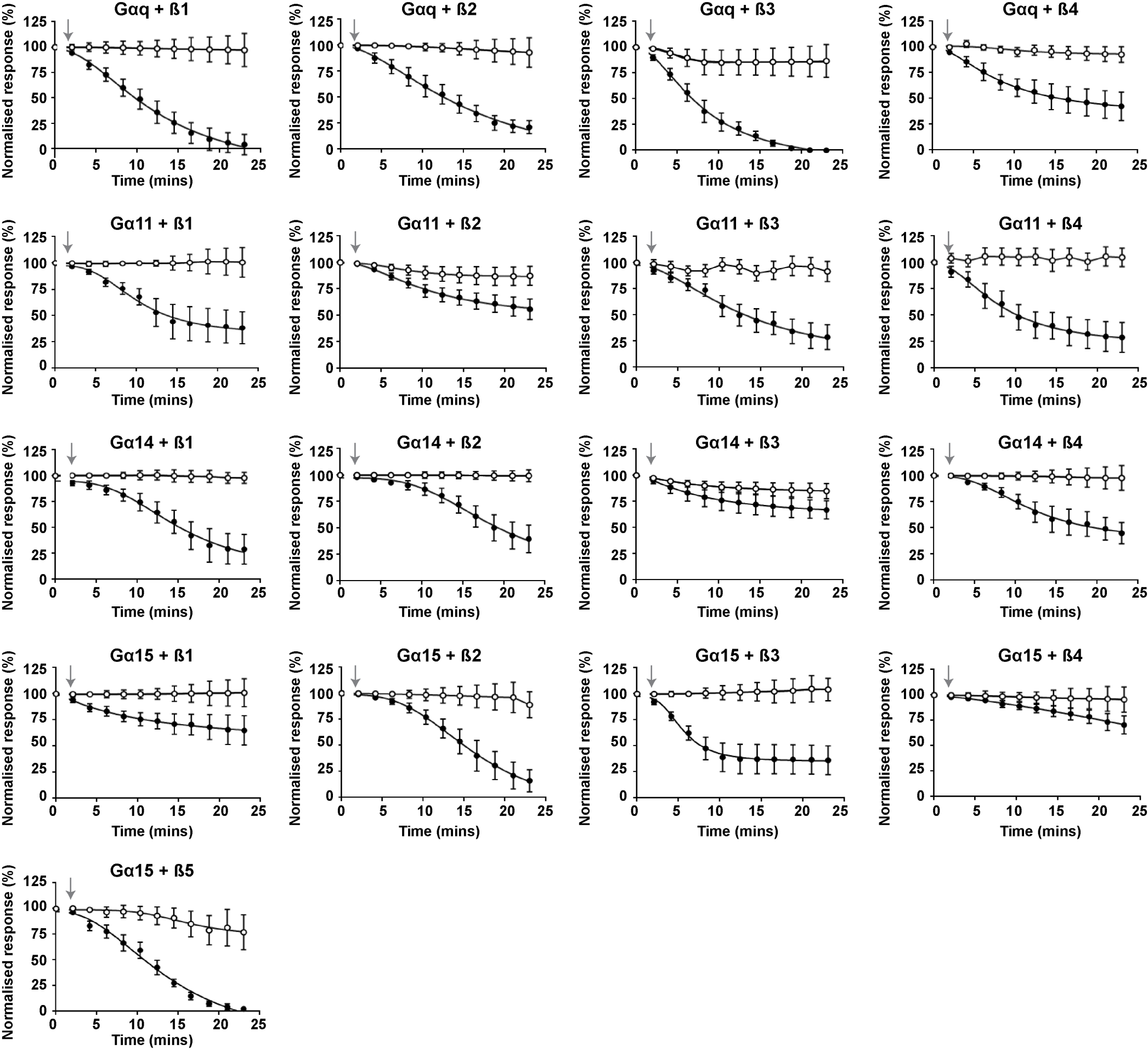

Supplement: Supplementary Figure 7 NanoBiT G-protein dissociation assays of the Gq/11 subfamily NanoBiT dissociation assays of AdHEK-CaSR cells transiently transfected with: LgBiT-Gα (q, 11, 14, 15), SmBiT-Gβ subunits (Gβ 1 - 5) and unlabelled Gγ2. Each panel shows dissociation when cells were exposed to 0.1mM  [file supplementary_figure_7.pdf]

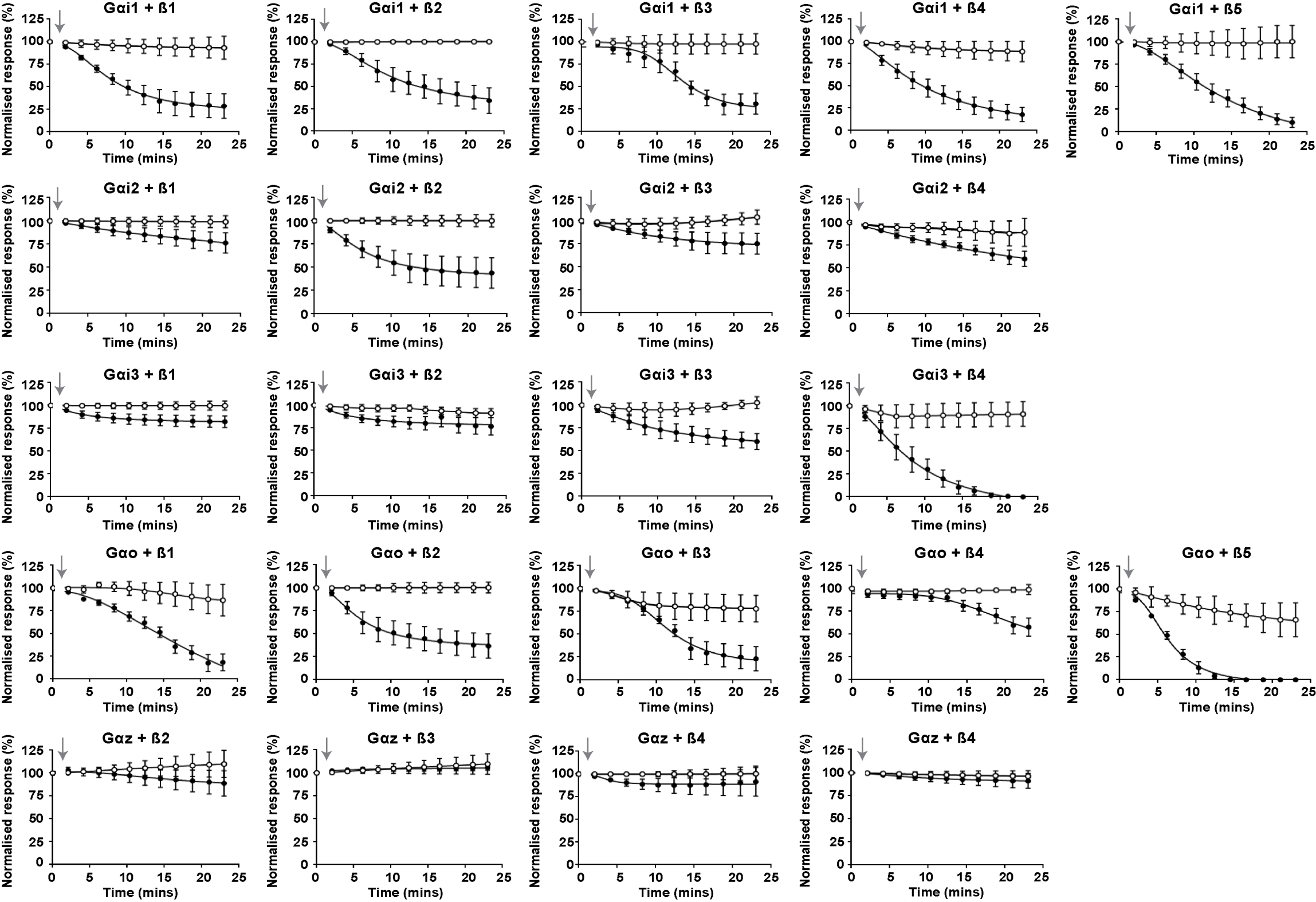

Supplement: Supplementary Figure 8 NanoBiT G-protein dissociation assays of the Gi/o subfamily NanoBiT dissociation assays of AdHEK-CaSR cells transiently transfected with: LgBiT-Gα (i1, i2, i3, o or z), SmBiT-Gβ subunits (Gβ 1 - 5) and unlabelled Gγ2. Each panel shows dissociation when cells were exposed to 0. [file supplementary_figure_8.pdf]

# SSTR5 + $G\alpha_z$ + $\beta_4$

Normalised response (%)

Agonist

○ Vehicle  
● SST

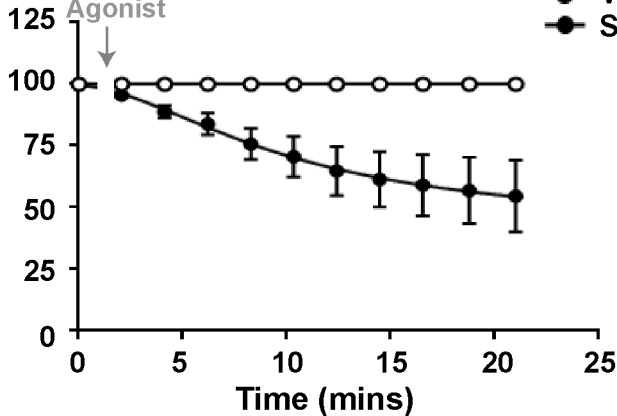

Supplement: Supplementary Figure 9 NanoBiT G-protein dissociation assay showing SSTR5 activates Gαz NanoBiT dissociation assays of AdHEK cells transiently transfected with: pcDNA-SSTR5, LgBiT-Gαz, SmBiT-Gβ4 and unlabelled Gγ2. Cells were exposed to vehicle (DMSO) or 50nM somatostatin (SST). Curves show mean±SEM [file supplementary_figure_9.pdf]

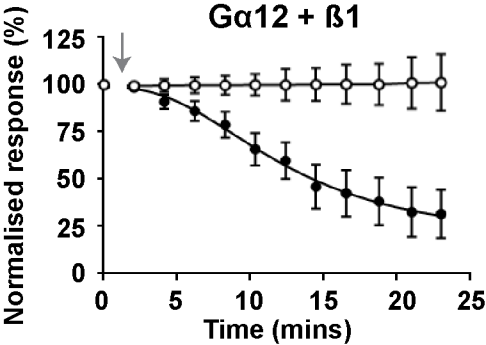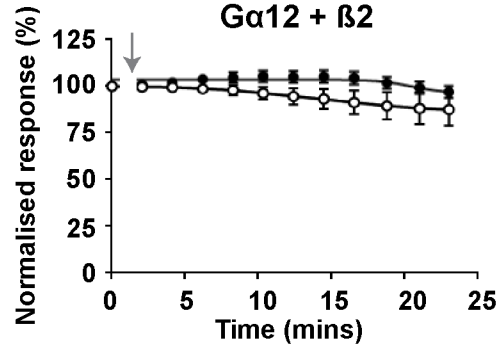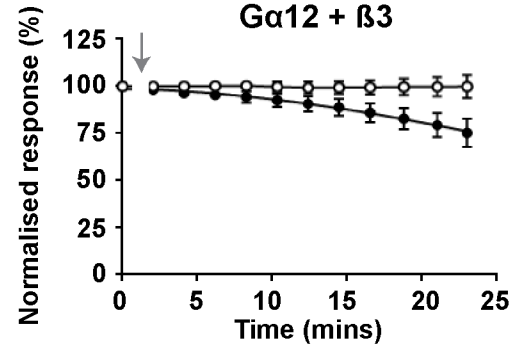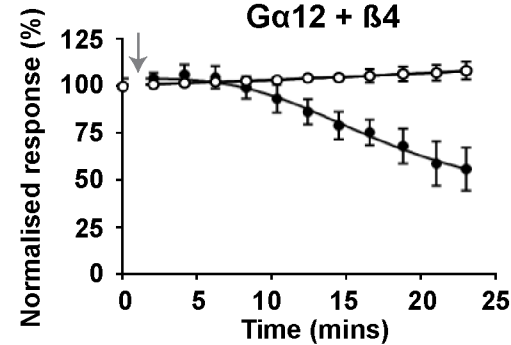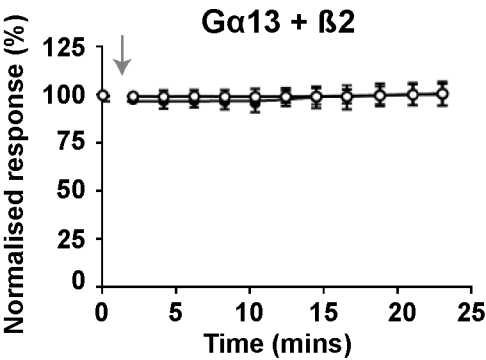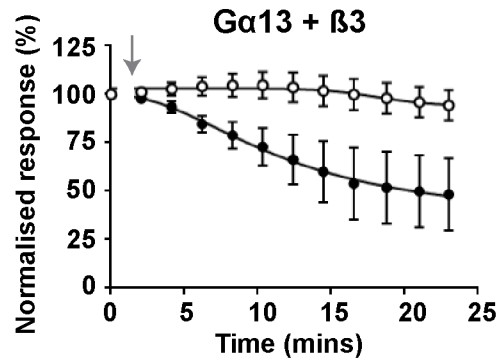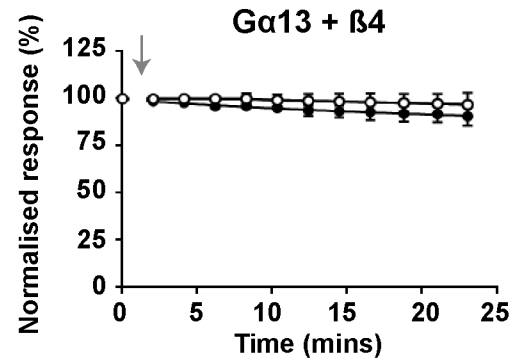

[CaCl<sub>2</sub>] mM

○ 0.1

● 5

Supplement: Supplementary Figure 10 NanoBiT G-protein dissociation assays of the G12/13 subfamily NanoBiT dissociation assays of AdHEK-CaSR cells transiently transfected with: LgBiT-Gα (12 or 13), SmBiT-Gβ subunits (Gβ 1 - 5) and unlabelled Gγ2. Each panel shows dissociation when cells were exposed to 0.1mM Ca2 [file supplementary_figure_10.pdf]

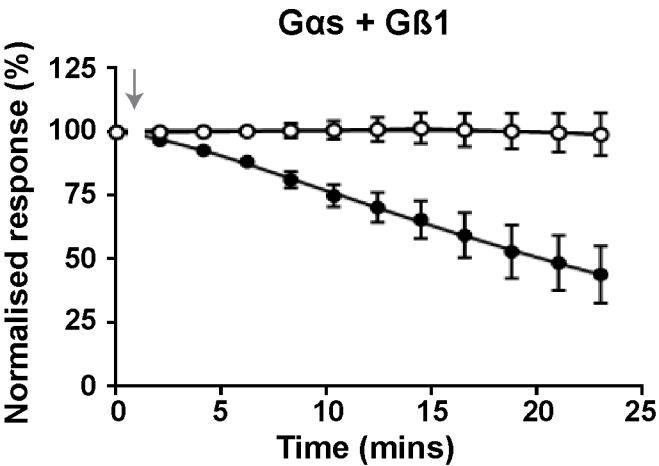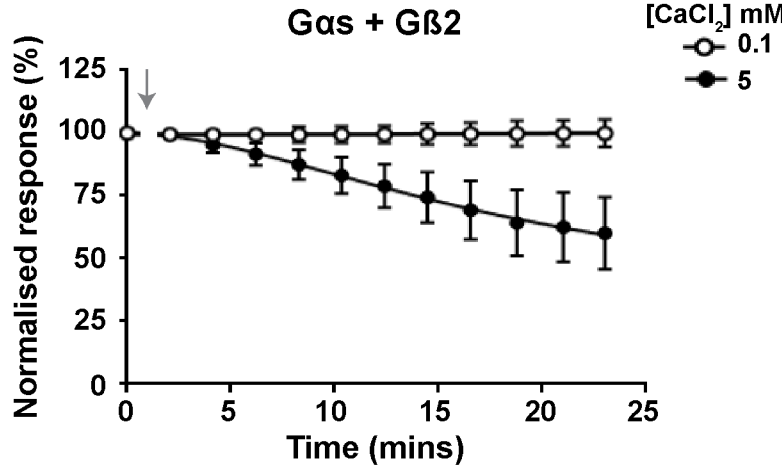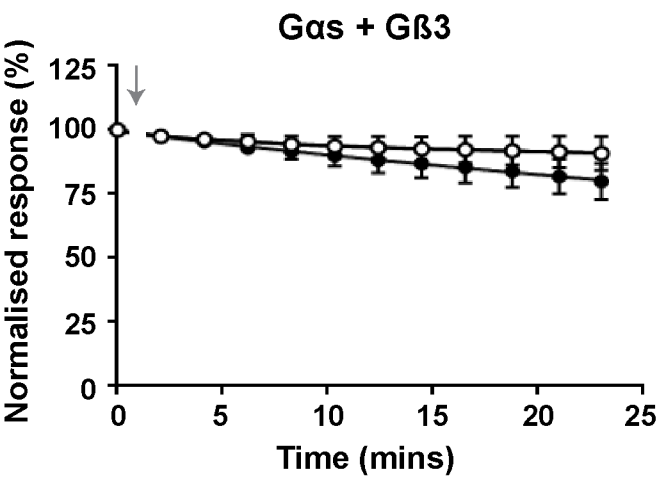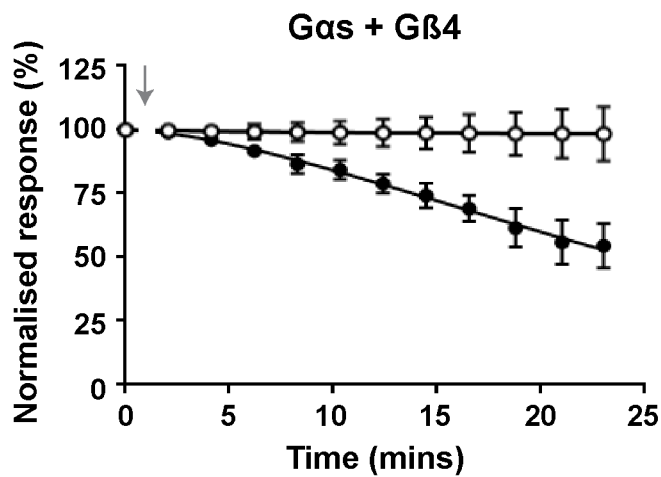

Supplement: Supplementary Figure 11 NanoBiT G-protein dissociation assays of the Gs/l subfamily NanoBiT dissociation assays of AdHEK-CaSR cells transiently transfected with: LgBiT-Gαs, SmBiT-Gβ subunits (Gβ 1 - 5) and unlabelled Gγ2. Each panel shows dissociation when cells were exposed to 0.1mM Ca2+e (open, wh [file supplementary_figure_11.pdf]
