# Supplementary material for: Heterogeneity of G protein activation by the calcium-sensing receptor
Source: J Mol Endocrinol. 2021 Jun 2;67(2):41–53. doi: 10.1530/JME-21-0058 (PMC8240730; doi:10.1530/JME-21-0058)
Supplement: Supplementary Table 1 Relative luminescence units for Gα-Gβγ pairings used in NanoBiT analyses [file supplementary_table_1.pdf]

Supplementary Table 1 Relative luminescence units for Gα-Gβγ pairings used in NanoBiT analyses

Gα	β1 mean ± SEM	β2 mean ± SEM	β3 mean ± SEM	β4 mean ± SEM	β5 mean ± SEM
**i1**	1429843 ± 9681	1538596.13 ± 20637	1517952.5 ± 14689	1477760 ± 102008	14368.75 ± 3279
**i2**	13961.81 ± 1900	343928.69 ± 81589	436015.69 ± 65129	66027.94 ± 20803	3243.5 ± 533
**i3**	29376.88 ± 4080	439990.38 ± 10326	486706.38 ± 76539	31343.25 ± 19999	<1000
**o**	255533.19 ± 6709	1467052.88 ± 21648	1640812.5 ± 95518	1579270.79 ± 143680	<1000
**s**	80423.88 ± 1977	224591.42 ± 77462	107235.75 ± 17669	78888.81 ± 13498	1769.38 ± 151
**z**	3465 ± 649	149008.88 ± 57452	205348.08 ± 15159	105112.33 ± 45407	<1000
**q**	12416 ±1846	95445.25 ± 17662	22129.44 ± 4918	34965.75 ± 8176	<1000
**11**	1624 ± 163	207794.61 ± 86590	65921.75 ± 8932	21793.71 ± 1510	<1000
**12**	1734.06 ± 72.47	164404.25 ± 79632	73417.06 ± 3417	13272.8 ± 4091	<1000
**13**	1360.38 ± 67.33	146960.06 ± 69571	149904.63 ± 13198	63529.92 ± 11665	<1000
**14**	4260.06 ± 1523	255160.71 ± 108754	179421 ± 20788	126477.06 ± 37195	<1000
**15**	1423.44 ± 141	349389.14 ± 13756	151675.5 ± 11980	43598.13 ± 11545	2224 ± 499

Expression of LgBiT-Gα with empty SmBiT and LgBiT empty with SmBiT-Gβ had <1000 relative luminescence units.
