# Supplementary material for: Heterogeneity of G protein activation by the calcium-sensing receptor
Source: J Mol Endocrinol. 2021 Jun 2;67(2):41–53. doi: 10.1530/JME-21-0058 (PMC8240730; doi:10.1530/JME-21-0058)
Supplement: Supplementary Table 2 Statistical analyses comparing % maximal suppression of baseline for NanoBiT dissociation assays [file supplementary_table_2.pdf]

**Supplementary Table 2 Statistical analyses comparing % maximal suppression of baseline for NanoBiT dissociation assays**

**Gq/11 family**

| Gq | p-value |
|---|---|
| b1 q vs. b4 15 | * |
| b1 q vs. b1 i2 | ** |
| b1 q vs. b3 i2 | ** |
| b1 q vs. b1 i3 | ** |
| b1 q vs. b2 i3 | ** |
| b1 q vs. b1 z | *** |
| b1 q vs. b2 z | **** |
| b1 q vs. b3 z | * |
| b1 q vs. b4 z | *** |
| b1 q vs. b2 12 | **** |
| b1 q vs. b3 12 | ** |
| b1 q vs. b2 13 | **** |
| b1 q vs. b4 13 | **** |
| b1 q vs. b2 s | * |
| b1 q vs. b3 s | ** |
| b2 q vs. b1 z | * |
| b2 q vs. b2 z | *** |
| b2 q vs. b4 z | * |
| b2 q vs. b2 12 | ** |
| b2 q vs. b2 13 | ** |
| b2 q vs. b4 13 | *** |
| b2 q vs. b3 s | * |
| b3 q vs. b2 11 | * |
| b3 q vs. b3 14 | * |
| b3 q vs. b1 15 | * |
| b3 q vs. b4 15 | ** |
| b3 q vs. b1 i2 | *** |
| b3 q vs. b3 i2 | *** |
| b3 q vs. b4 i2 | * |
| b3 q vs. b1 i3 | *** |
| b3 q vs. b2 i3 | *** |
| b3 q vs. b3 i3 | * |
| b3 q vs. b4 o | * |
| b3 q vs. b1 z | **** |
| b3 q vs. b2 z | **** |
| b3 q vs. b3 z | *** |
| b3 q vs. b4 z | **** |
| b3 q vs. b2 12 | **** |
| b3 q vs. b3 12 | *** |
| b3 q vs. b2 13 | **** |
| b3 q vs. b4 13 | **** |
| b3 q vs. b2 s | ** |
| b3 q vs. b3 s | **** |

| G11 | p-value |
|---|---|
| b3 11 vs. b2 z | ** |
| b3 11 vs. b2 12 | * |
| b3 11 vs. b2 13 | * |
| b3 11 vs. b4 13 | ** |
| b4 11 vs. b2 z | ** |
| b4 11 vs. b2 12 | * |
| b4 11 vs. b2 13 | * |
| b4 11 vs. b4 13 | ** |

| G14 | p-value |
|---|---|
| b1 14 vs. b2 z | * |
| b1 14 vs. b2 12 | * |
| b1 14 vs. b4 13 | * |
| b2 14 vs. b2 z | * |

| G15 | p-value |
|---|---|
| b2 15 vs. b2 z | ** |
| b2 15 vs. b2 12 | * |
| b2 15 vs. b2 13 | * |
| b2 15 vs. b4 13 | * |
| b3 15 vs. b2 z | * |
| b3 15 vs. b2 12 | * |
| b3 15 vs. b4 13 | * |
| b4 15 vs. b5 15 | ** |
| b4 15 vs. b5 i1 | * |
| b4 15 vs. b4 i3 | * |
| b4 15 vs. b5 o | * |
| b5 15 vs. b1 i2 | *** |
| b5 15 vs. b3 i2 | ** |
| b5 15 vs. b1 i3 | *** |
| b5 15 vs. b2 i3 | ** |
| b5 15 vs. b1 z | *** |
| b5 15 vs. b2 z | **** |
| b5 15 vs. b3 z | ** |
| b5 15 vs. b4 z | *** |
| b5 15 vs. b2 12 | **** |
| b5 15 vs. b3 12 | ** |
| b5 15 vs. b2 13 | **** |
| b5 15 vs. b4 13 | **** |
| b5 15 vs. b2 s | ** |
| b5 15 vs. b3 s | *** |

**Gi/o family**

| Gi1 | p-value |
|---|---|
| b1 i1 vs. b2 z | ** |
| b1 i1 vs. b2 12 | * |
| b1 i1 vs. b2 13 | * |
| b1 i1 vs. b4 13 | * |
| b2 i1 vs. b2 z | * |
| b2 i1 vs. b4 13 | * |
| b3 i1 vs. b1 z | * |
| b3 i1 vs. b2 z | *** |
| b3 i1 vs. b4 z | * |
| b3 i1 vs. b2 12 | ** |
| b3 i1 vs. b2 13 | ** |
| b3 i1 vs. b4 13 | *** |
| b4 i1 vs. b1 i2 | * |
| b4 i1 vs. b1 i3 | * |
| b4 i1 vs. b1 z | * |
| b4 i1 vs. b2 z | **** |
| b4 i1 vs. b4 z | ** |
| b4 i1 vs. b2 12 | *** |
| b4 i1 vs. b2 13 | *** |
| b4 i1 vs. b4 13 | *** |
| b4 i1 vs. b3 s | * |
| b5 i1 vs. b1 i2 | ** |
| b5 i1 vs. b3 i2 | ** |
| b5 i1 vs. b1 i3 | ** |
| b5 i1 vs. b2 i3 | ** |
| b5 i1 vs. b1 z | *** |
| b5 i1 vs. b2 z | **** |
| b5 i1 vs. b3 z | ** |
| b5 i1 vs. b4 z | *** |
| b5 i1 vs. b2 12 | **** |
| b5 i1 vs. b3 12 | ** |
| b5 i1 vs. b2 13 | **** |
| b5 i1 vs. b4 13 | **** |
| b5 i1 vs. b2 s | * |
| b5 i1 vs. b3 s | *** |

| Gi2 | p-value |
|---|---|
| b1 i2 vs. b4 i3 | ** |
| b1 i2 vs. b5 o | * |
| b2 i2 vs. b2 z | * |
| b3 i2 vs. b4 i3 | ** |
| b3 i2 vs. b5 o | * |

| Gi3 | p-value |
|---|---|
| b1 i3 vs. b4 i3 | ** |
| b1 i3 vs. b5 o | * |
| b2 i3 vs. b4 i3 | * |
| b2 i3 vs. b5 o | * |
| b4 i3 vs. b1 z | ** |
| b4 i3 vs. b2 z | **** |
| b4 i3 vs. b3 z | * |
| b4 i3 vs. b4 z | ** |
| b4 i3 vs. b2 12 | **** |
| b4 i3 vs. b3 12 | ** |
| b4 i3 vs. b2 13 | **** |
| b4 i3 vs. b4 13 | **** |
| b4 i3 vs. b3 s | ** |

| Go | p-value |
|---|---|
| b1 o vs. b1 z | * |
| b1 o vs. b2 z | *** |
| b1 o vs. b4 z | * |
| b1 o vs. b2 12 | ** |
| b1 o vs. b2 13 | ** |
| b1 o vs. b4 13 | ** |
| b1 o vs. b3 s | * |
| b2 o vs. b2 z | * |
| b3 o vs. b2 z | *** |
| b3 o vs. b4 z | * |
| b3 o vs. b2 12 | ** |
| b3 o vs. b2 13 | ** |
| b3 o vs. b4 13 | ** |
| b5 o vs. b1 z | ** |
| b5 o vs. b2 z | **** |
| b5 o vs. b3 z | * |
| b5 o vs. b4 z | ** |
| b5 o vs. b2 12 | *** |
| b5 o vs. b3 12 | * |
| b5 o vs. b2 13 | *** |
| b5 o vs. b4 13 | *** |
| b5 o vs. b3 s | ** |

| Gz | p-value |
|---|---|
| b2 z vs. b1 12 | ** |

**G12/13 family**

| G12 | p-value |
|---|---|
| b1 12 vs. b2 12 | * |
| b1 12 vs. b2 13 | * |
| b1 12 vs. b4 13 | * |

Analyses were performed in Graphpad using one-way ANOVA. Only comparisons that were significantly different are shown in the table.
