# Supplementary material for: Heterogeneity of G protein activation by the calcium-sensing receptor
Source: J Mol Endocrinol. 2021 Jun 2;67(2):41–53. doi: 10.1530/JME-21-0058 (PMC8240730; doi:10.1530/JME-21-0058)
Supplement: Supplementary Table 3 Statistical analyses comparing expression of G-protein α-subunits in human parathyroid tissue [file supplementary_table_3.pdf]

Supplementary Table 3 **Statistical analyses comparing expression of G-protein α-subunits in human parathyroid tissue**

	GNAQ	***GNA11***	***GNA14***	***GNA15***	***GNA12***	***GNA13***	***GNAS1***	***GNAL***	***GNAI1***	***GNAI2***	***GNAI3***	***GNAO1***	***GNAZ***
GNAQ		**	NS	**	NS	NS	NS	NS	NS	NS	NS	****	NS
GNA11			***	****	*	NS	*	*	NS	*	*	****	NS
GNA14				NS	NS	NS	NS	NS	NS	NS	NS	**	*
GNA15					*	**	*	*	***	NS	*	*	****
GNA12						NS	NS	NS	NS	NS	NS	**	NS
GNA13							NS	NS	NS	NS	NS	***	NS
GNAS1								NS	NS	NS	NS	*	NS
GNAL									NS	NS	NS	*	NS
GNAI1										NS	NS	****	NS
GNAI2											NS	**	NS
GNAI3												***	NS
GNAO1													****
GNAZ													

Statistical analyses of data shown in Figure 4A. Analyses were performed by one-way ANOVA. ****p<0.0001, ***p<0.001, **p<0.01, *p<0.05, NS – not significant.
