# Supplementary material for: Heterogeneity of G protein activation by the calcium-sensing receptor
Source: J Mol Endocrinol. 2021 Jun 2;67(2):41–53. doi: 10.1530/JME-21-0058 (PMC8240730; doi:10.1530/JME-21-0058)
Supplement: Supplementary Table 4 Statistical analyses comparing expression of G-protein α-subunits in human kidney tissue [file supplementary_table_4.pdf]

Supplementary Table 4 **Statistical analyses comparing expression of G-protein α-subunits in human kidney tissue**

	GNAQ	*GNA11*	*GNA14*	*GNA15*	*GNA12*	*GNA13*	*GNAS1*	*GNAL*	*GNAI1*	*GNAI2*	*GNAI3*	*GNAO1*	*GNAZ*
GNAQ		NS	NS	NS	NS	NS	****	NS	NS	NS	NS	NS	NS
GNA11			NS	NS	NS	NS	****	NS	NS	NS	NS	NS	NS
GNA14				NS	NS	NS	****	NS	NS	NS	NS	NS	NS
GNA15					NS	NS	****	NS	NS	NS	NS	NS	NS
GNA12						NS	****	NS	NS	NS	NS	NS	NS
GNA13							****	NS	NS	NS	NS	NS	NS
GNAS1								****	****	****	****	****	****
GNAL									NS	NS	NS	NS	NS
GNAI1										NS	NS	NS	NS
GNAI2											NS	NS	NS
GNAI3												NS	NS
GNAO1													NS
GNAZ													

Statistical analyses of data shown in Figure 6A. Analyses were performed by one-way ANOVA. ****p<0.0001, NS – not significant.
