# Supplementary material for: Heterogeneity of G protein activation by the calcium-sensing receptor
Source: J Mol Endocrinol. 2021 Jun 2;67(2):41–53. doi: 10.1530/JME-21-0058 (PMC8240730; doi:10.1530/JME-21-0058)
Supplement: Supplementary Table 5 Statistical analyses comparing expression of G-protein α-subunits in human pancreatic tissue [file supplementary_table_5.pdf]

Supplementary Table 5 Statistical analyses comparing expression of G-protein α-subunits in human pancreatic tissue

	GNAQ	***GNA11***	***GNA14***	***GNA15***	***GNA12***	***GNA13***	***GNAS1***	***GNAL***	***GNAI1***	***GNAI2***	***GNAI3***	***GNAO1***	***GNAZ***
GNAQ		NS	NS	NS	NS	NS	****	NS	NS	NS	NS	NS	NS
GNA11			NS	*	NS	NS	****	*	NS	NS	NS	NS	NS
GNA14				NS	NS	NS	****	NS	NS	NS	NS	NS	NS
GNA15					NS	NS	****	NS	NS	NS	NS	NS	NS
GNA12						NS	****	NS	NS	NS	NS	NS	NS
GNA13							****	NS	NS	NS	NS	NS	NS
GNAS1								****	****	****	****	****	****
GNAL									NS	NS	NS	NS	NS
GNAI1										NS	NS	NS	NS
GNAI2											NS	NS	NS
GNAI3												NS	NS
GNAO1													NS
GNAZ													

Statistical analyses of data shown in Figure 7A. Analyses were performed by one-way ANOVA. ****p<0.0001, NS – not significant.
